# Review Article: Synthesis, properties, and applications of fluorescent diamond particles

**DOI:** 10.1116/1.5089898

**Published:** 2019-04-12

**Authors:** Olga A. Shenderova, Alexander I. Shames, Nicholas A. Nunn, Marco D. Torelli, Igor Vlasov, Alexander Zaitsev

**Affiliations:** 1 Adámas Nanotechnologies, 8100 Brownleigh Dr., Raleigh, North California 27617; 2 Department of Physics, Ben-Gurion University of the Negev, Be’er-Sheva 8410501, Israel; 3 General Physics Institute, RAS, Vavilov Street 38, 119991 Moscow, Russia; 4 College of Staten Island, CUNY, 2800 Victory Blvd., Staten Island, New York 10312

## Abstract

Diamond particles containing color centers—fluorescent crystallographic defects embedded within the diamond lattice—outperform other classes of fluorophores by providing a combination of unmatched photostability, intriguing coupled magneto-optical properties, intrinsic biocompatibility, and outstanding mechanical and chemical robustness. This exceptional combination of properties positions fluorescent diamond particles as unique fluorophores with emerging applications in a variety of fields, including bioimaging, ultrasensitive metrology at the nanoscale, fluorescent tags in industrial applications, and even potentially as magnetic resonance imaging contrast agents. However, production of fluorescent nanodiamond (FND) is nontrivial, since it requires irradiation with high-energy particles to displace carbon atoms and create vacancies—a primary constituent in the majority color centers. In this review, centrally focused on material developments, major steps of FND production are discussed with emphasis on current challenges in the field and possible solutions. The authors demonstrate how the combination of fluorescent spectroscopy and electron paramagnetic resonance provides valuable insight into the types of radiation-induced defects formed and their evolution upon thermal annealing, thereby guiding FND performance optimization. A recent breakthrough process allowing for production of fluorescent diamond particles with vibrant blue, green, and red fluorescence is also discussed. Finally, the authors conclude with demonstrations of a few FND applications in the life science arena and in industry.

## I. INTRODUCTION

**Fig. 1.**
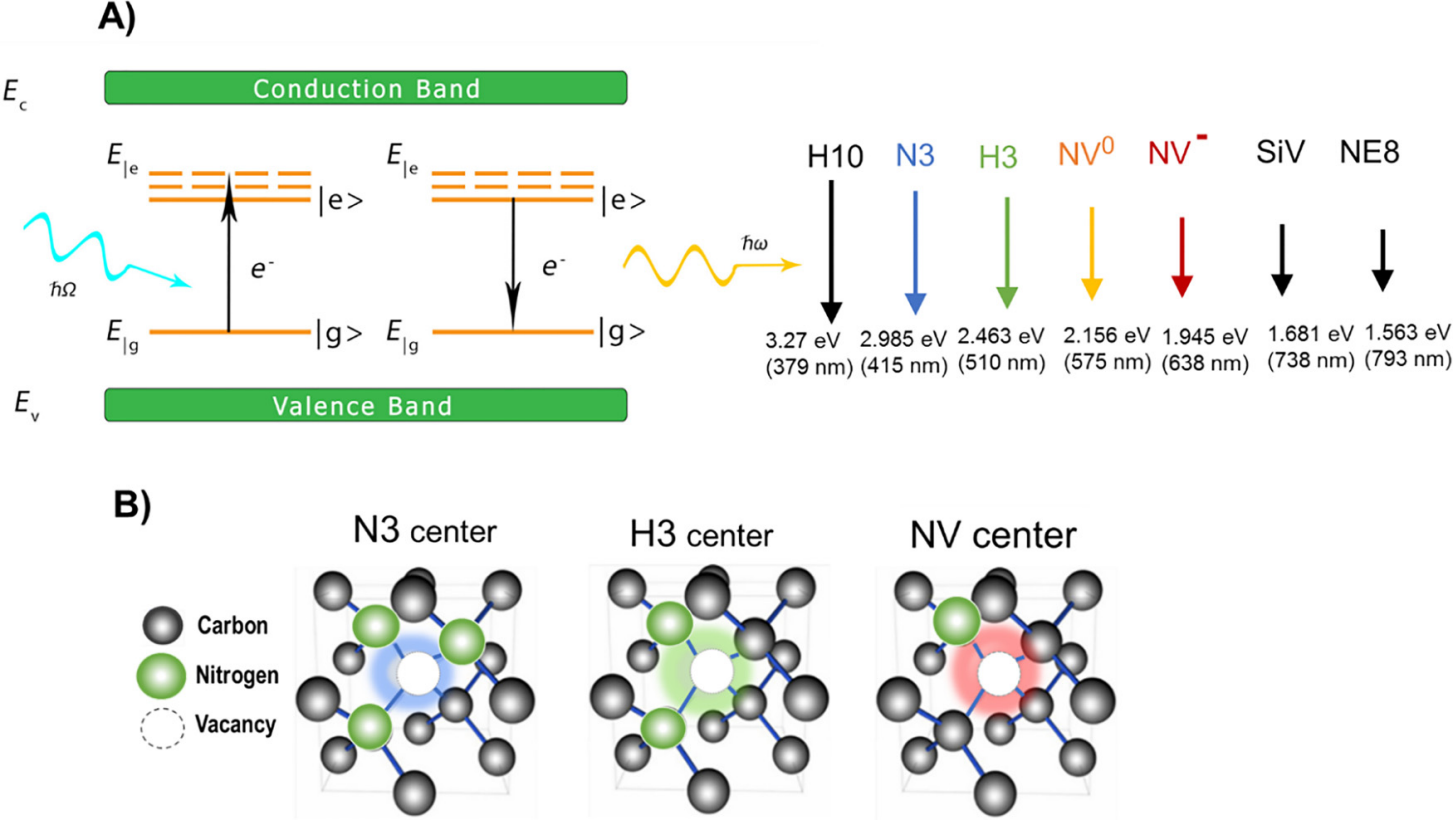
(a) Schematics of energy states of typical luminescent crystallographic defects in diamond with well-defined ground and excited energy states located within the large bandgap of diamond (5.49 eV). The array of centers identified on the right illustrates the broad range of luminescence which occurs from defects of different origin, ranging from the UV, through the visible, to the NIR region of the spectrum. Energies and corresponding wavelengths attributed to the zero-phonon lines of the defects are provided. N3, H3, and NV centers are nitrogen-related centers emitting in the visible spectrum; the H10 center emitting in the UV is attributed to an electronic transition to an excited state of the H3 center (Ref. 1); centers emitting in the NIR are the SiV (a silicon-vacancy center) and NE8 (a complex consisting of Ni atom surrounded by 4 N atoms and 2 vacancies) (Ref. 1). (b) Crystallographic structures of N3, H3, and NV centers composed of complexes of a vacancy and nitrogen atoms. NV centers can be negatively charged (NV^−^) or have a neutral charge (NV^0^).

Presently, hundreds of different luminescent centers are known to exist in diamond^1,2^ and new ones continue to be discovered.^3–7^ This extensive variety of luminescent centers—a feature less prevalent in other minerals—results from diamond’s unique chemical structure and physical properties.^1^ First, diamond has the highest known atomic density of all solids, consisting of carbon atoms in a tetrahedral arrangement forming a dense network of very short and strong covalent bonds. Therefore, any foreign atom incorporated into the diamond lattice strongly interacts with the electronic structure of the carbon atoms and introduces highly localized electronic states in the diamond band gap. Absorption of light by such defects results in excitation of electrons from one well-defined energy state to another and emission of photons upon relaxation of the electron to the ground state. Second, the large bandgap of diamond (5.49 eV) is favorable for luminescence because the radiative electronic transitions require that both the ground and excited states lie within the bandgap [Fig. 1(a)]. Third, diamond has the highest Debye temperature of any solid (∼2000 K) so that phonon excitation occurs at elevated temperatures and phonon-electron coupling does not disturb electronic transitions from the ground to the excited state of the optical defects up to relatively high temperatures. For example, NV^0^ and H3 defects exhibit luminescence even above 500 °C.^1^ In addition, diamond possesses the widest optical transparency window of all known solids (from 0.22 *μ*m to the far-infrared) so that selected defects exhibit luminesce in the ultraviolet (UV), visible, and IR spectral regions, while the high mechanical hardness and thermal conductivity of diamond contribute to optical characteristics with unmatched photostability. Optically active defects of various origin and structure exist in diamond, including intrinsic and impurity-related and point or extended defects. Many impurities are known to form optically active defects in diamond including H, He, Ne, Xe, Li, B, N, O, P, Si, Ge, As, Ti, Cr, Ni, Co, Zn, Zr, Ag, W, and Tl, with the majority of these elements being introduced via ion implantation.^1^ Most optically active defects in diamond are complexes consisting of impurity atoms bound to intrinsic structural defects (vacancies and/or carbon interstitials). Of all defects, nitrogen-based defects produce the broadest array of luminescent centers (more than 50),^2^ extending through the entire visible spectrum as well as into the near-infrared (NIR) [Fig. 1(b)]. Nitrogen is a native impurity in diamond and is of special importance; for example, the physical classification of diamond is based primarily upon the optical absorption and concentration of nitrogen.^2^

Investigation of color centers in diamond is an area of active research in both the gemological community^2,8^ and the nanotechnology arena.^9–12^ In the gemological world, efforts are directed toward spectroscopic analysis of various treatments of natural diamonds which can be performed to improve their optical clarity or induce enhanced coloration. Treatment of natural diamond by methods such as the high-pressure high-temperature (HPHT) process have been developed to improve the transparency of natural diamond and increase their price.^2^ HPHT treatment is also accompanied by reduced fluorescence of colorless diamond, which is valuable, since fluorescence reduces the value of colorless diamond and about 30% of natural diamonds are fluorescent.^13^ For production of desirable, fancy-colored gems, defect centers created by radiation damage and their evolution upon thermal annealing are of particular interest.^14–18^ Focused studies into these topics began as early as the 1950s.^14^ Since all treatments of natural diamond must be disclosed to buyers, identification of spectroscopic fingerprints that reveal undisclosed treatments for artificial coloration and improved transparency is one focus of diamond gemological studies.^2^ Another important arena of gemological research is identification of gems of undisclosed synthetic origin, since color centers in man-made diamond are unique from those in natural diamond. The necessity for such studies emerged as a result of recent success in the growth of large single crystal diamonds using the chemical vapor deposition (CVD) technique, which permits the production of both colorless and fancy-colored artificial gemstones upon irradiation/thermal annealing.^2^

While the majority of fundamental optical studies in diamond have been performed on bulk diamond,^1,14–18^ controlled production of color centers in particulate diamond, and specifically in nanosized diamond, has become an area of active research due to emerging applications in quantum information processing,^9^ bioimaging,^10,19^ and nanosensing.^11,12^ The exceptional photostability and biocompatibility of fluorescent nanosized diamond particles makes them particularly attractive in specific bioimaging applications requiring imaging agents to be stable under high power laser irradiation, such as in superresolution imaging^20^ or to provide a reliable fluorescent signal over a prolonged period of time, such as in image guided surgery.^21^ In addition to stable fluorescence, the negatively charged nitrogen-vacancy color center (NV^−^) possesses an unpaired electronic spin, a property that has significant implications for a number of applications, including high precision measurements of environmental parameters such as electromagnetic field,^22^ temperature,^23,24^ and mechanical strain.^25^ Similar to other fluorescent materials, fluorescent diamond particulate (nano- and micro-sized) can be used in a wide range of industrial applications, including fluorescent paints and coatings,^26–28^ security inks and forensic tracers,^26,29^ fluid tracers,^26^ and solar energy concentrators for photovoltaics,^30^ to name a few. Competitive advantages of diamond-based fluorescent agents include their mechanical and chemical robustness, photo- and thermal stability, and environmental safety due to the absence of toxicity.

While fluorescent diamond particles (FDPs) are emerging as unique fluorophores in a variety of applications, fluorescent nanodiamond (FND) production is challenging, since it requires irradiation with high-energy atomic or subatomic particles to displace carbon atoms in order to create vacancies. This review is primarily focused on material developments. Key steps in the production of FND are discussed, with emphasis on the current challenges facing the field and material and possible solutions. Until recently, commercial production of fluorescent diamond particulate was mostly centered around FDPs with red/NIR emission based on nitrogen-vacancy (NV) centers.^10,31–33^ To be competitive with other fluorophores, production of FDPs with emission at other wavelengths is required. We discuss how the combination of controlled atomic doping of diamond, controlled creation of radiation defects (vacancies), and controlled formation of defect and dopant complexes via thermal treatment *allows* the production of FDPs with vibrant blue, green, and red fluorescence. Special attention is paid to the use of electron paramagnetic resonance (EPR) as a valuable instrument for the assessment of paramagnetic defects in FDPs, providing valuable information on the types of radiation defects formed and their evolution upon thermal annealing. In combination with fluorescent spectroscopy, this method provides valuable insights for FDP performance optimization. Finally, we conclude the review with demonstrations of several FDP applications in the life sciences and in industry.

## II. PRODUCTION AND CHARACTERIZATION OF FLUORESCENT DIAMOND PARTICLES

**Fig. 2.**
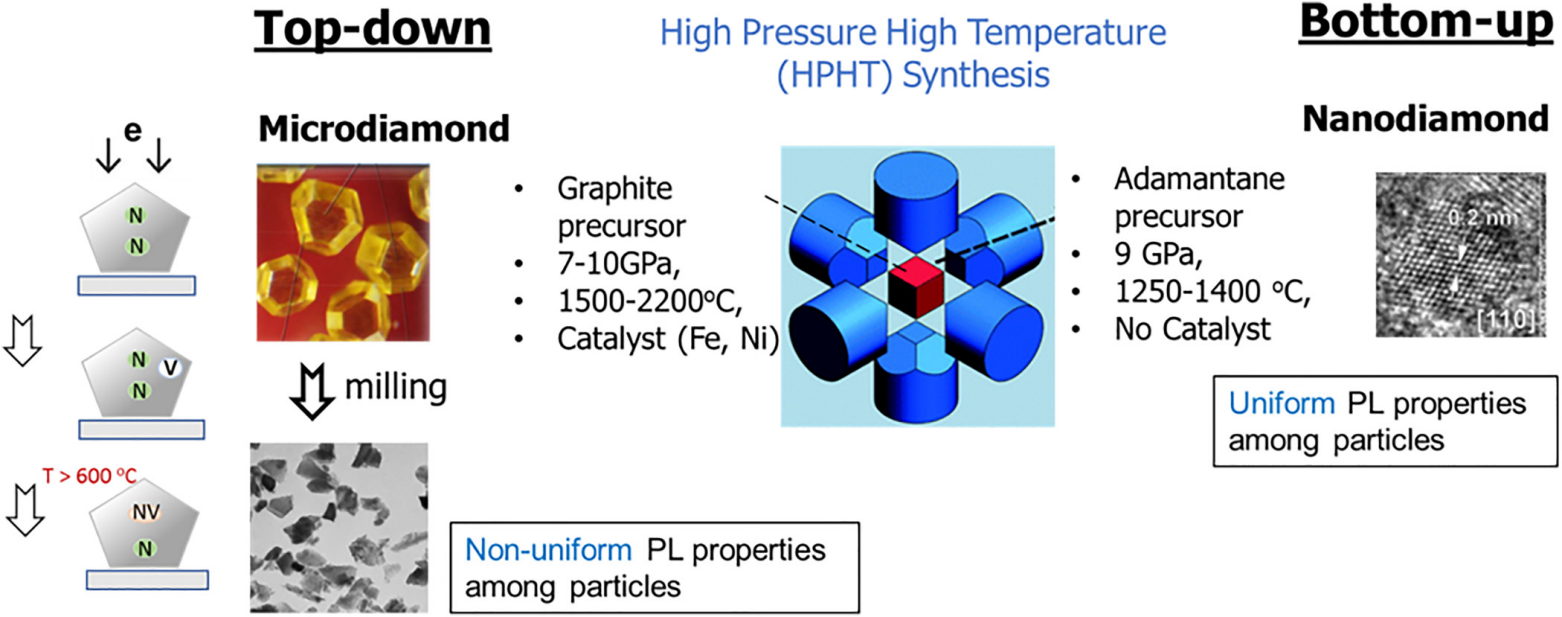
Schematic of HPHT growth of micron-sized particles of type Ib and their processing to smaller particles (top-down approach) with formation of NV centers by irradiation and annealing (in either micron-sized particles before milling or in a postmilling process). Alternatively, the HPHT method can be used for synthesis of submicron sized particles with more uniform dopant atom distribution in the precursor material resulting in higher control of uniformity of fluorescence among particles (bottom-up approach). Figure adapted with permission from N. Nunn, M. Torelli, G. McGuire, and O. Shenderova, Curr. Opin. Solid State Mater. Sci. **21**, 1 (2017). Copyright 2017, Elsevier (Ref. 132).

Commercial production of diamond particles containing NV centers is currently based on the use of diamond particles synthesized by the HPHT method. Hydraulic presses are used to generate pressures of 7–10 GPa at temperatures of 1500–2200 °C to produce diamond from a graphitic precursor in the presence of metal catalysts (Fe, Ni, or/and Co) [Fig. 2(a)].^34^ HPHT diamond particles with sizes exceeding 100 *μ*m containing 100–200 ppm of substitutional nitrogen (type Ib) are typically produced. These particles are then milled to fractions of smaller sizes. The micron-sized particles (1 *μ*m and above) and the submicron particles (∼100 nm–1 *μ*m) are widely used in the abrasives industry. Commercial quantities of nanosized diamond with sizes of 100 nm and below containing NV centers have been produced by irradiation of these particles with He ions^10^ or 1–3 MeV electrons.^32^ More robust micron-sized diamond particles are used when a high fluence and/or high flux are needed, the latter translates into reduced cost of irradiation. Micron-sized particles have been used for commercial production of fluorescent diamond particles using electron beams with energies 10 MeV,^31^ 5 MeV,^33^ or 1–3 MeV.^32^ These micron-sized HPHT particles containing color centers are then milled to nanosized particles [Fig. 2(a)] by what can be classified as a top-down method (Fig. 2). The top-down approach has benefits and limitations. Since the production of abrasive diamond grit of HPHT origin is a >60 year old industry, the material is very affordable (∼$2/g) and is produced in tons of quantities annually. However, the nitrogen content and distribution in these particles is not optimized, since these particles are primarily used in the abrasives industry where control of nitrogen content is not important. A major drawback of using these HPHT diamond particles to produce nanosized FND particles is that the nitrogen atoms are heterogeneously distributed. Nitrogen incorporation into the crystal is dependent on the crystallographic orientation of the growth surface, where (100) diamond facets are depleted of nitrogen. The nitrogen concentration in synthetic diamond crystals also decreases with increasing radius of a diamond crystal due to the depletion of nitrogen over time in the metallic catalyst melt.^35^ As a result, powder obtained by crushing of HPHT microdiamond to smaller sizes exhibits a heterogeneous nitrogen spatial distribution in different particles (Fig. 2). Nonuniform nitrogen distribution among crushed particles results in nonuniform photoluminescence. One option to overcome this challenge is to use a bottom-up approach to synthesize nanosized diamond particles with controlled nitrogen (and other dopants) content, including an approach of HPHT synthesis of nanodiamond (ND) from nontraditional carbon precursors (Fig. 2). Major bottom-up approaches which are currently at an exploratory stage are briefly discussed below, followed by a detailed discussion of the major steps used in the current, commercial, top-down synthesis of nanodiamond particles containing NV (ND-NV) centers.

### A. Bottom-up synthesis of diamond nanoparticles containing color centers

#### 1. New class of HPHT diamond

**Fig. 3.**
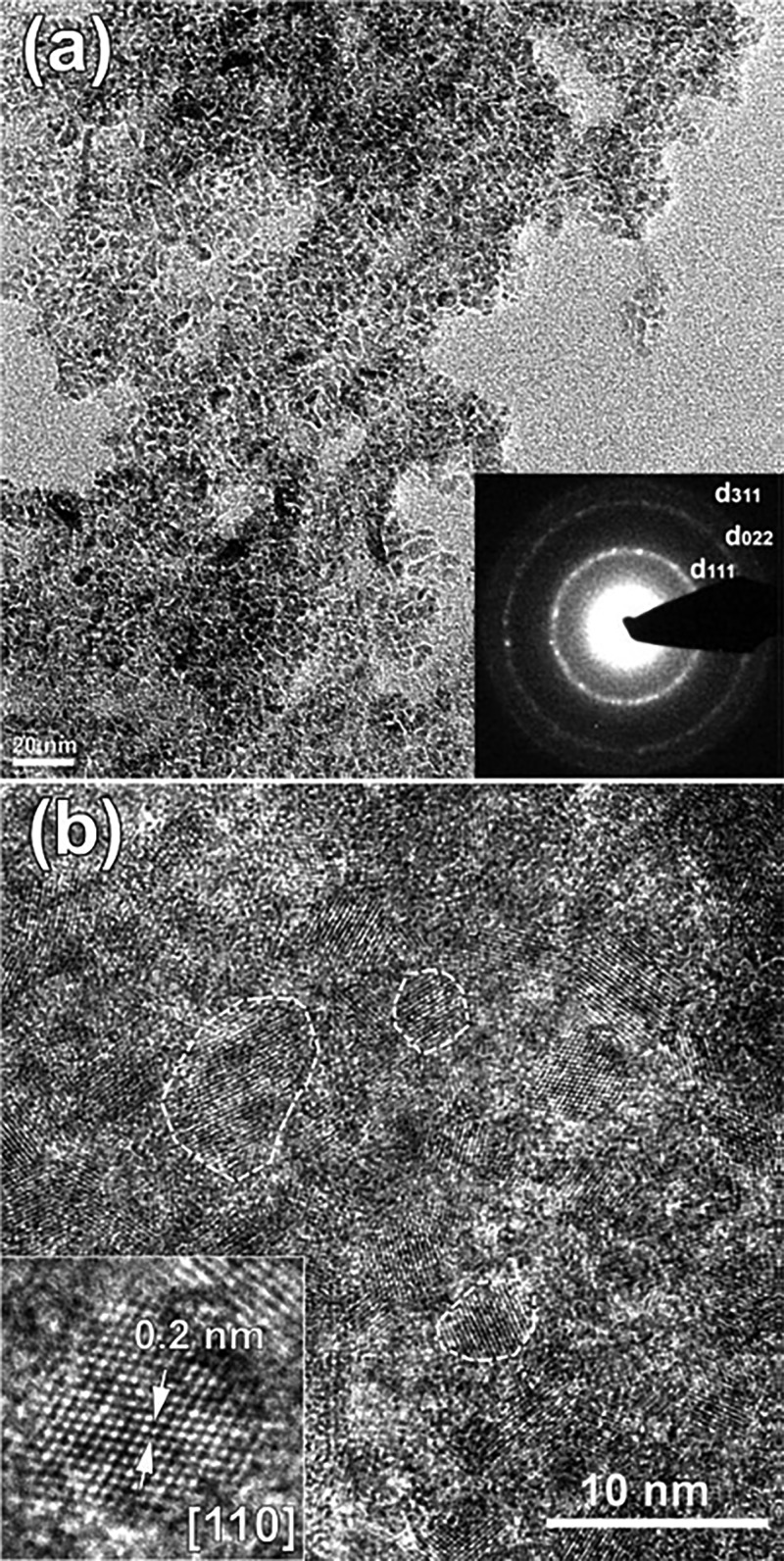
(a) TEM image of diamond nanoparticles, inset: the corresponding ring electron diffraction pattern is evidence of the diamond structure, (b) HRTEM of selected area in (a). Some of the nanoparticles depicted with dash line. Inset: enlarged HRTEM image of a single diamond particle about 3 nm in size. Reproduced with permission from E. A. Ekimov, O. S. Kudryavtsev, N. E. Mordvinova, O. I. Lebedev, and I. I. Vlasov, ChemNanoMat. **4**, 269 (2018). Copyright 2018, John Wiley & Sons, Inc.

A new class of diamond materials, nano- and micro-diamond particles synthesized from organic compounds using the HPHT technique, has been under development in recent years.^12,36–39^ Particles are produced under thermodynamically stable conditions that are the most favorable for obtaining perfect diamond structures and do not contain metal impurities inherent for traditional HPHT synthesis where metallic media are used. Due to the high structural quality of the diamond matrix, it was possible to obtain nanodiamond particles containing SiV and GeV centers with narrow luminescence lines when synthesized from hydrocarbons in the presence of silicon and germanium dopants.^40,41^ The possibility of nanodiamond synthesis from its molecular analog, adamantane, was also demonstrated.^39^ According to Raman analysis, adamantane starts to transform into diamond in a titanium capsule at a pressure of about 9 GPa and a temperature above 1250 °C. A nanocrystalline form of diamond is dominant in the temperature range from 1250 to 1400 °С, whereas at higher temperatures, diamond microcrystals are primarily formed.^42^ Diamond crystals with sizes down to 3 nm (Fig. 3) were produced at a synthesis temperature of 1330 °C.^39^

**Fig. 4.**
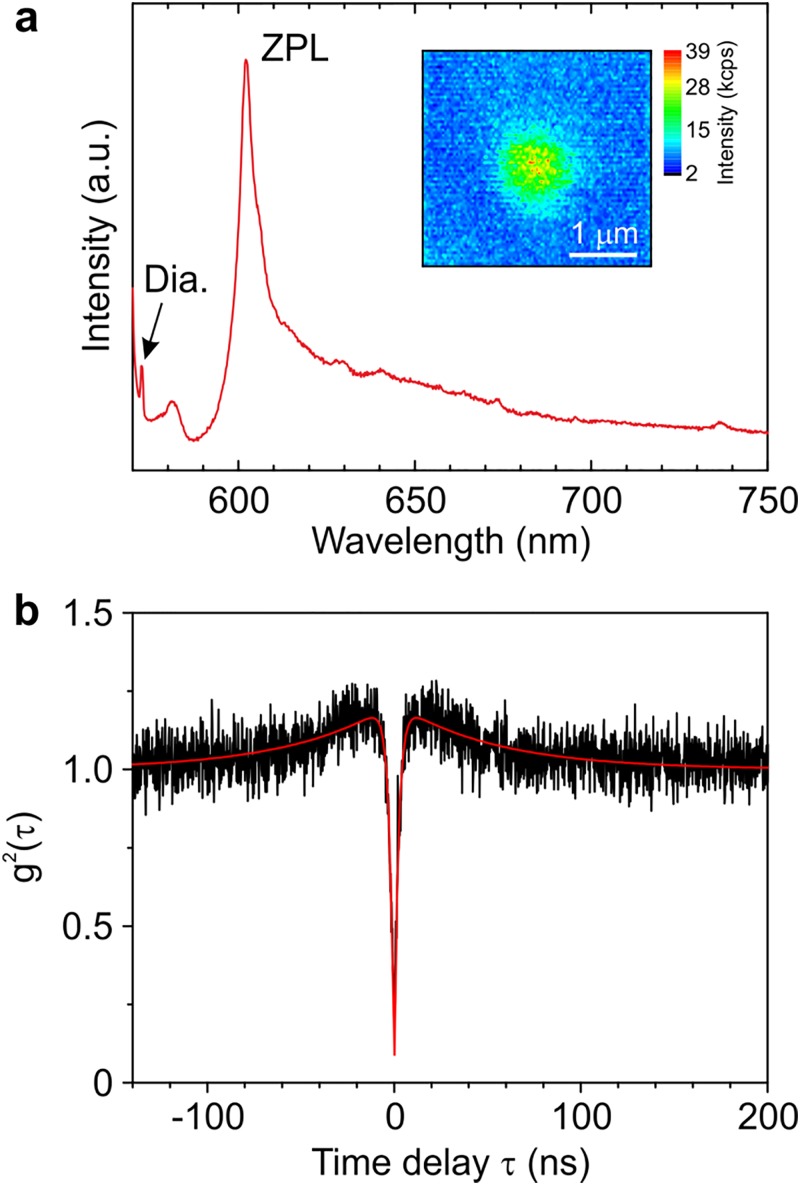
GeV single photon emitters in nanodiamonds produced from a mixture of adamantane and tetraphenylgermane. (a) PL spectrum of isolated crystallite. Inset: confocal fluorescence microscope image of this crystallite. (b) Second-order autocorrelation function. The red curve denotes the fitting. The measurements were performed at room temperature. Reprinted with permission from E. A. Ekimov, M. V. Kondrin, V. S. Kriyobok, A. V. Khomich, I. I. Vlasov, R. A. Khmelnitskiy, T. Iwasaki, and M. Hatano, Diam. Relat. Matter (in press). Copyright 2019, Elsevier.

**Fig. 5.**
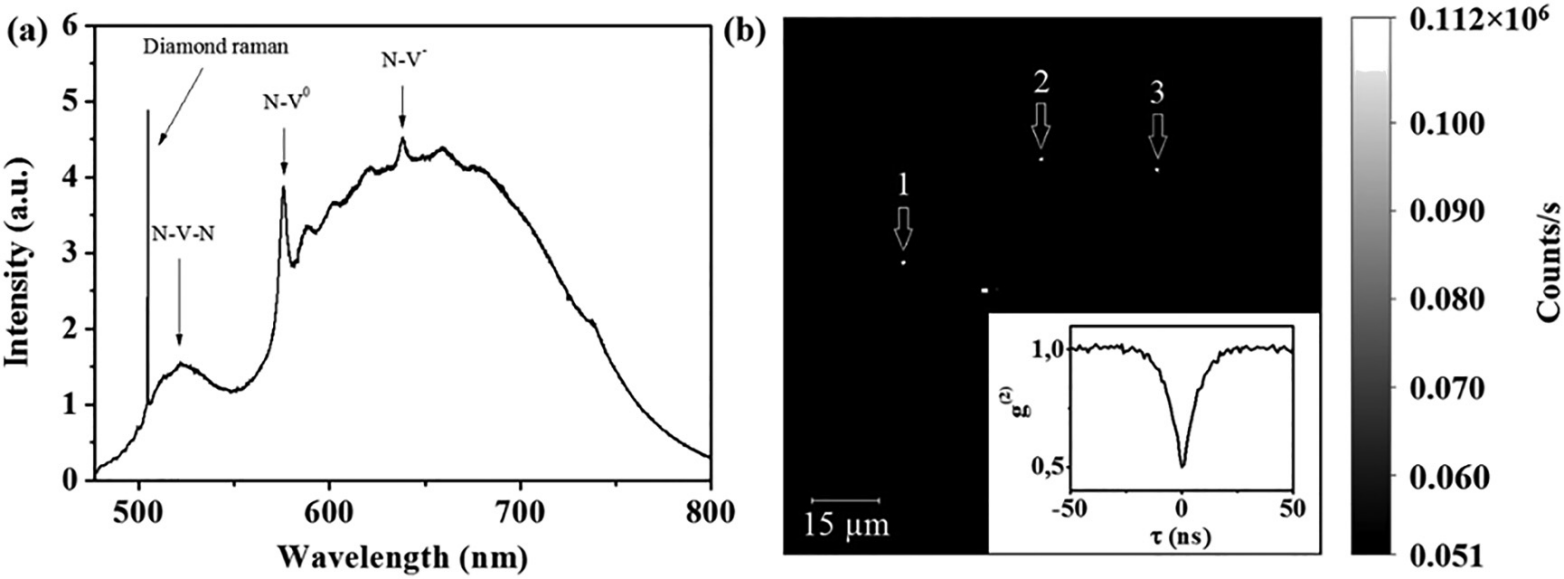
NV^−^ single photon emitters in nanodiamonds produced from a mixture of adamantane and adamantane carbonitrile. (a) PL spectrum of diamond powder. (b) Confocal fluorescence microscope image of isolated diamond particles, three crystallites with sizes of about 200 nm are numbered as 1–3. Inset: Second-order autocorrelation function, measured for one of these crystallites. The measurements were performed at room temperature.

The possibility of doping “organic” diamonds with optically active impurities is demonstrated by the synthesis of nano- and micro-diamonds, containing nitrogen-, silicon-, and germanium-related centers.^39,43^ Controlling the concentration of dopants in organic precursors allows one to produce diamond crystallites containing one luminescent center. For instance, decreasing the concentration of Ge down to 0.004 at. % in the mixture of adamantane with C_24_H_20_Ge, 50-nm diamonds with a single GeV center were obtained.^43^ Figure 4(a) shows the GeV luminescence image for one such diamond nanoparticle. Measurement of second-order autocorrelation function, g^2^(τ), for the GeV emission confirms single photon emission from this particle [Fig. 4(b)]. Larger crystallites were synthesized from a mixture of adamantane and adamantane carbonitrile. Their sizes ranged from 200 up to 2 *μ*m. A typical photoluminescence spectrum for the sample produced at an N/C ratio of 0.02 at. % in the precursor is shown in Fig. 5(a). The photoluminescence lines from three types of nitrogen-related defects are observed in the spectrum: (1) “double nitrogen-vacancy” (NVN or H3) centers, (2) neutrally charged “nitrogen-vacancy” centers (NV^0^), and (3) negatively charged “nitrogen-vacancy” centers (NV^−^). Luminescence of these centers is characterized by narrow zero-phonon lines (ZPLs) at 504 nm (H3), 575 nm (NV^0^), and 638 nm (NV^−^), which are accompanied by a set of broad lines (phonon replicas) at shorter wavelengths. For these samples, 1–3 NV^−^ centers were detected in most of the smallest (200–300 nm) crystallites [Fig. 5(b)]. Good uniformity in the distribution of nitrogen and, as a result, of NV centers in the volume of the sample is achieved by premixing the original organic compounds in a liquid solvent.

#### 2. CVD synthesis of nanodiamonds

**Fig. 6.**
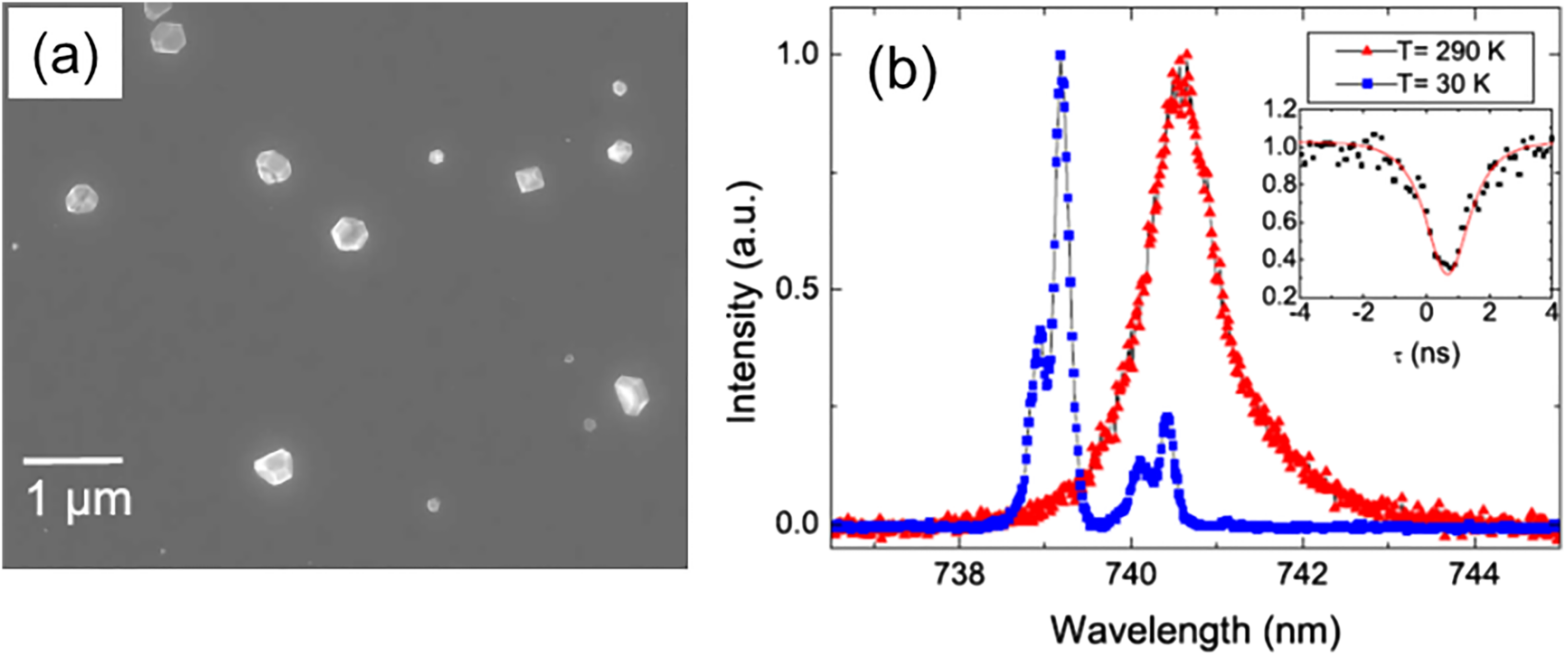
(a) SEM images of CVD nanodiamonds. E. Neu, C. Hepp, M. Hauschild, S. Gsell, M. Fischer, H. Sternschulte, D. Steinmüller-Nethl, M. Schreck, and C. Becher, New J. Phys. **15**, 043005 (2013); licensed under a Creative Commons Attribution (CC BY) license (Ref. 133). (b) Temperature-dependent spectra of SiV emitter. The spectra have been measured at 290 K (red triangles) and 30 K (blue squares). The inset displays the g^2^ function of the emitter. E. Neu, D. Steinmetz, J. Riedrich-Möller, S. Gsell, M. Fischer, M. Schreck, and C. Becher, New J. Phys. **13**, 025012 (2011); licensed under a Creative Commons Attribution (CC BY) license (Ref. 134).

**Fig. 7.**
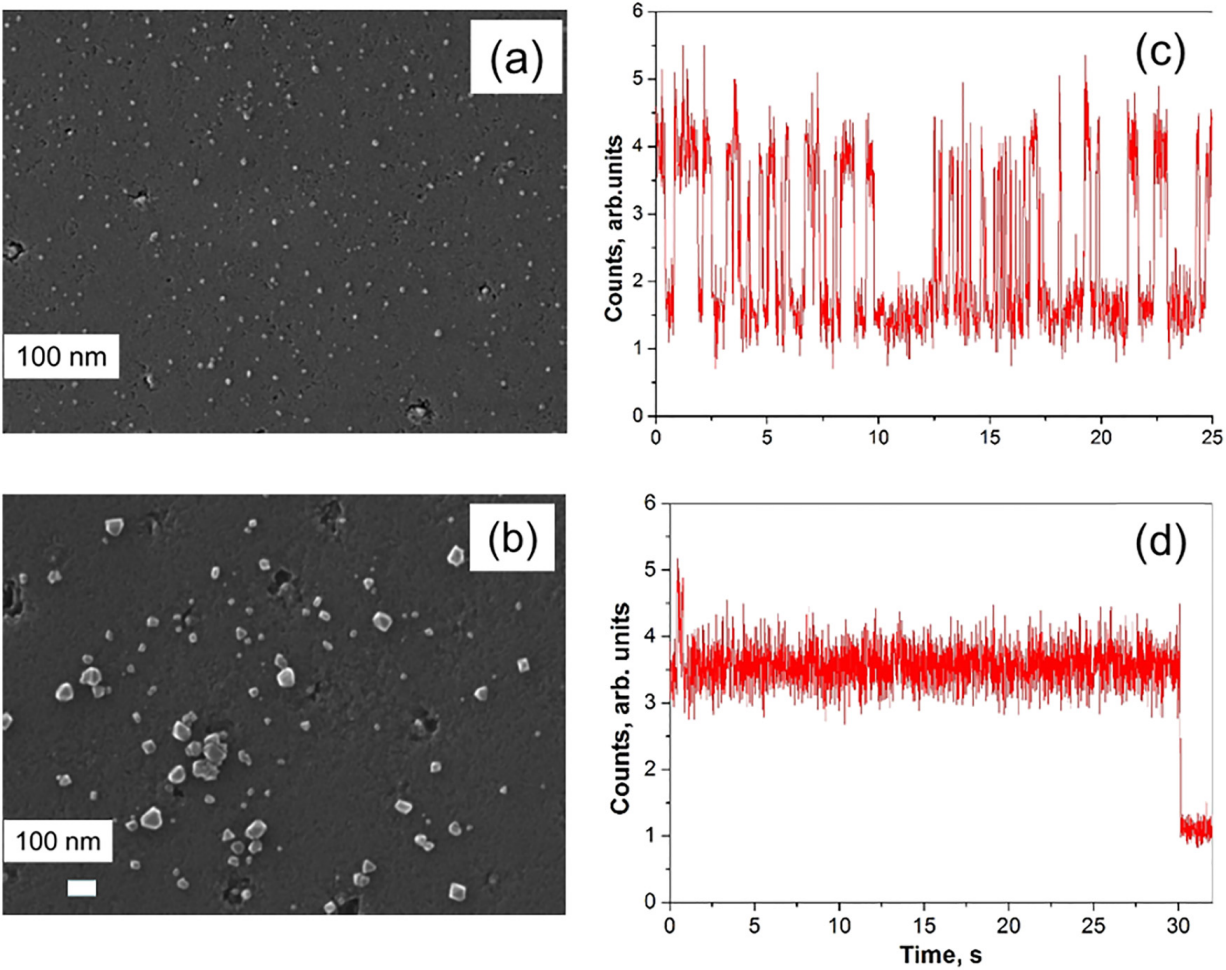
SEM images of diamond particles with a mean thickness of the CVD diamond layer of 2 nm (a) and 12 nm (b). Typical time traces of NV luminescence for particles, shown in (a) and (b), are plotted in (c) and (d), respectively. The excitation laser beam position was shifted from the emitting particle at 30 s to demonstrate the level of background signal (d). (c) and (d) reproduced with permission from V. A. Shershulin, V. S. Sedov, A. Ermakova, U. Jantzen, L. Rogers, A. A. Huhlina, E. G. Teverovskaya, V. G. Ralchenko, F. Jelezko, I. I. Vlasov, Phys. Status Solidi. A **212**, 2600–2605 (2013). Copyright 2013, John Wiley & Sons, Inc. (Ref. 135).

CVD is another technique used for “bottom-up” synthesis of nanodiamonds; however, it does not allow mass production of nanodiamonds. According to our estimates, one cycle of CVD diamond synthesis (typically about 1 h) enables one to produce ∼0.01 mg of 100 nm-diamond particles. The advantages of this method are that no additional purification of synthesized NDs is required, and nanocrystals are grown with a preselected density of luminescent centers and location on the substrate, which facilitates studying the optical and spin properties of the luminescent centers in individual crystallites. CVD grown crystals with a mean size of 130 nm [Fig. 6(a)] were deposited on an irridium substrate seeded with synthetic nanodiamonds (Microdiamant Liquid Diamond MSY, Switzerland) with sizes up to 30 nm.^44^ The individual crystals were studied by luminescence spectroscopy at cryogenic temperatures. SiV color centers were identified in CVD nanodiamonds, and measurement, for the first time, of the fine structure splitting of a single SiV center was achieved [Fig. 6(b)]. In Kudryavtsev *et al.*,^45^ the dependence of SiV and NV luminescence on CVD crystal size was studied. The nanodiamonds produced consisted of an HPHT diamond core (Tomei Diamond Co., Japan) with a mean size of 20 nm and an epitaxial CVD diamond outer layer of different thicknesses up to 12 nm [Figs. 7(a) and 7(b)]. The proximity of the surface suppresses the NV luminescence and causes photo-blinking [Figs. 7(c) and 7(d)]. The interaction of the luminescent centers with the nanodiamond surface was shown to become insignificant at a distance to its surface exceeding 12 nm for NV centers and 4 nm for SiV centers.

### B. Top-down commercial production of diamond particles containing NV centers

A number of groups are currently undertaking efforts to commercialize ND-NV centers.^31–33,46^ Recently, our group succeeded in large-scale production of fluorescent diamond particles containing NV centers in hundred-grams per batch scales using irradiation with 2–3 MeV electrons.^32^ Production of ND-NV fractions with median sizes ranging between 10 and 100 nm was achieved by milling of micron-sized particles and subsequent purification; Fluorescent nanodiamonds with different types of surface modification and functionalization are now available as commercial products.^32^ In this section, we summarize both our groups’ findings and insights from other groups toward optimization of NV centers production in diamond particles.

#### 1. Major steps for production of nanodiamonds containing NV centers

**Fig. 8.**
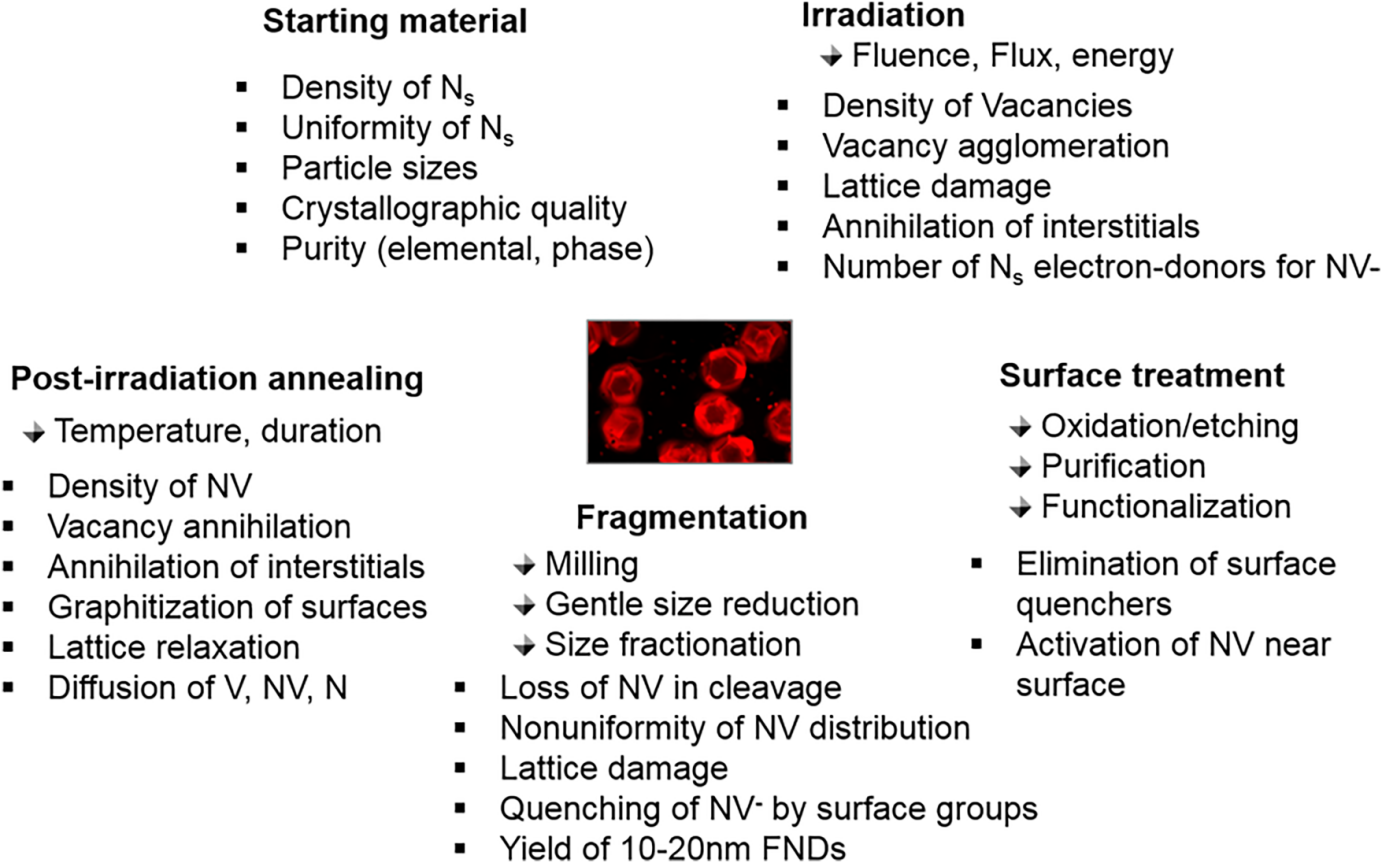
Summary of factors influencing production of fluorescent color centers in diamond particles containing NV centers. Abbreviations: NV, Nitrogen-Vacancy; V, Vacancy; N_s_, Substitutional nitrogen.

Figure 8 summarizes the major factors influencing color center production in diamond particles. In order to produce fluorescent particles, it is first necessary to form vacancies in HPHT type Ib diamond powders. Vacancies can be created by irradiation with high-energy electrons (1–10 MeV),^31–33,47–49^ protons (2–3 MeV),^49,50^ low energy (40 keV) alpha-particles,^46^ gamma rays,^51,52^ and fast neutrons.^53^ Several factors must be considered regarding the choice of the type of irradiation and the overall strategy of NV centers production, such as production efficiency, uniformity of vacancies production along the radiation path, vacancy survival rate after irradiation, the overall lattice damage resulting from irradiation, the amount of diamond material treated in a single run, and availability of the irradiation source. All irradiation methods have some advantages and disadvantages. Irradiation with He^+^ produces many vacancies per ion; therefore, the fluence required for a specified vacancy concentration is very low (∼10^13^ ions/cm^2^).^46^ Fluences employed in proton irradiation of diamonds vary from 10^15^ to 10^16^ p/cm^2^.^48^ Irradiation with electrons is not as efficient in terms of the number of the vacancies produced per electron (e.g., ∼2 vacancies/e^−^ for 5 MeV electrons^51^ versus 13 and 40 vacancies created per 3 MeV H^+^ and He^+^ ions, respectively^46^); however, this can be partially compensated by the greater availability of electron beams with currents up to 3 orders of magnitude higher than those for He^+^ ion beams. Typical electron irradiation fluencies currently employed for the production of NV centers in various diamond samples range from approximately 10^18^ to 5 × 10^19^ e^−^/cm^2^,^31–33^ with a few research groups reporting fluences as high as 10^20^ e^−^/cm^2^.^33^

While heavier ions are more efficient in terms of production of vacancies per particle, irradiation with the former is often a cause of extended lattice damage, resulting in amorphization of a portion of the lattice along the path of the radiation cascade. For example, the maximum achieved brightness in particles was almost twofold higher for irradiation with protons as compared to heavier He^+^ ions at approximately the same theoretical density of vacancies generated,^54^ presumably due to lower lattice damage produced by protons. With just a few vacancies produced per electron, lower overall lattice damage is expected using electron beam irradiation. Furthermore, an e-beam based radiation procedure results in a more homogeneous distribution of vacancies versus those created along the short radiation track of a larger irradiating species. For example, recent studies have shown that the distribution of NV centers along the path of a proton in a millimeter-sized diamond crystal is highly nonuniform.^55^ The distribution of vacancies created by an He^+^ beam is more uniform than upon irradiation with protons,^46^ but still less uniform than obtained by irradiation with electrons.^51^ The irradiation penetration depth for 40 keV He^+^ and 3 MeV H^+^ ions is short: 200 nm and 50 *μ*m, respectively.^46^ Irradiation with 5 MeV electrons provides for much longer—up to 7 mm—penetration into the diamond,^51^ thus increasing the amount of material which may be treated in a single run. However, at high e-beam fluences, the lattice damage can also start to accrue and contribute to decreased efficiency of NV formation.

Irradiation with fast neutrons (>2000-fold heavier than electrons) is another efficient approach for vacancies production on a large scale (potentially a kilogram in a single run) and at fluences lower than required for electron beams.^53^ However, the induced radioactivity, particularly from metal catalyst impurities left within the diamond lattice during synthesis, is a major hurdle preventing wide scale adaptation of this method. Recently though, an interesting approach for NV formation was demonstrated using light ions formed homogeneously *in situ* by a nuclear reaction.^56^ Nanodiamond particles embedded in B_2_O_3_ were irradiated by neutrons. Neutrons captured by ^10^B generated an isotropic flux of energetic α particles and ^7^Li+ ions that uniformly irradiated the surrounding nanodiamonds and produced vacancies. Due to the high production yield of this approach, it has good commercial perspectives.

To summarize, electron beam irradiation is considered to be the most efficient in terms of NV center quality and production scales—both essential considerations for commercial production. Electron beam parameters such as energy, flux, and fluence all play important roles in the number of NV centers formed and their brightness. Interestingly, it was recently shown by Capelli *et al.* that temperature during electron irradiation can play a significant role in the density of nitrogen-vacancy color centers formed.^57^ Table I summarizes results of our irradiation of 100 nm HPHT type Ib diamond particles at different conditions.

**Table I.** Summary of characteristics of 100 nm HPHT diamond particles milled before irradiation and then irradiated with electrons at different irradiation conditions (samples 1–3). Sample 4 is 100 nm ND-NV obtained by milling of 15 *μ*m particles containing 8 ppm of NV centers. Electron irradiation conditions listed for sample 4 are related to the 15 *μ*m particles. Concentration of negatively charged NV centers is defined from EPR analysis. Photoluminescent brightness was measured in 1 mg/ml diamond suspensions in deionized water with 532 nm laser excitation.

Sample	Electron energy (MeV)	Fluence (e^−^/cm^2^)	Flux (relative unit)	Concentration of NV^−^ (ppm)	Brightness (relative units)
1	3	5 × 10^18^	1	3.0	1.5
2	3	1 × 10^19^	10	2.5	1.3
3	10	1 × 10^19^	…	2.1	1
4	3	2 × 10^19^	10	4.5	2.3

As can be seen from Table I, at a moderate fluence (5 × 10^18^ e^−^/cm^2^), 3 MeV electrons produced brighter particles than 10 MeV electrons. However, this result was obtained at lower flux (longer irradiation time). It should be noted, though, that the brightest sample from the series of 100 nm particles (samples 1–3) was irradiated under conditions which resulted in the sample temperature rising high enough to cause *in situ* annealing and subsequently NV centers formation. In addition, for this sample, the targeted fluence was achieved not in a single run, but over several irradiations with intermediate annealing to form NV centers from the vacancies induced during the previous run before performing the next irradiation.^58^

The next step after creation of vacancies is annealing of the irradiated powder at a temperature exceeding approximately 700 °C to evoke diffusion of vacancies and formation of NV centers. According to systematic annealing studies by Havlik *et al.*, the optimal annealing regime is 900 °C for 1 h.^59^

Yields of material during irradiation, annealing, and purification from nondiamond carbon differ dramatically for micron-sized and submicron sized particles. While the losses are only a few percent for 15 *μ*m particles, they can be up to 30%–50% for submicron particles. As such, the use of micron-sized diamond particles for production of ND-NV by milling is more commercially appealing. In addition, 100 nm-diamond particles obtained by milling of 15 *μ*m particles have higher brightness than ND-NV produced by direct irradiation of 100 nm particles (Table I). Still, the optimal parameter space is very complex and comprised of at least a dozen known factors that need to be taken into consideration (Fig. 8). Optimization of NV center production is based on developing an understanding of the underlying physical reasons for high brightness that can be tuned during processing. We systematically characterized NV center formation and the accompanying defects as a function of various processing parameters using a combination of EPR and photoluminescent spectroscopy.^60,61^ EPR is an important technique which provides the concentration of negatively charged NV centers in diamond, the major goal of processing.

#### 2. Optimization of NV centers formation

In this section, we discuss the roles of irradiation fluence, particle size, and initial nitrogen content in the starting particles as they relate to the efficiency of NV center production and the related luminescence brightness. Due to the importance of EPR findings as a means to explain the observed results, we start with an introductory section of the origin of EPR defects in diamond.

##### a. Origin of paramagnetic defects in diamonds

The phenomenon of EPR (also called electron spin resonance) is the resonant absorption of electromagnetic radiation by unpaired electron spins subjected to an external static magnetic field. Both a deep theoretical background and application of EPR to studies of a large variety of systems may be found in classic books by Weil and Bolton^62^ and Abragam/Bleaney.^63^ This nondestructive method provides researchers and technologists the potential to uniquely identify the electron-nuclear structure of various substances containing paramagnetic species, from gases to complex biological molecules. Each substance containing intrinsic and/or impurity atoms and molecules with unpaired electrons falls within the scope of EPR analysis, including complexes of transition and rare earth ions, free radicals, singlet and triplet irradiation-induced defects, magnetic gases, etc. EPR is capable of providing important information on the structure and dynamics of a system, including the chemical and physical processes occurring there.

EPR and related techniques (like electron nuclear double resonance, etc.) applied to studies of defects and impurities in crystalline diamond samples provide useful information on the anisotropy of electronic *g* tensor; zero-field couplings in species with *S* > 1/2; hyperfine interaction with principle nuclei of surrounding atoms, etc.^63,64^ Traditionally, EPR and related phenomena are interpreted within the framework of the spin-Hamiltonian (SH) concept, where SH is an energy operator describing all existing interactions (electric, magnetic, exchange) of the electron and nuclei spins with their surrounding and external fields applied.^62,63,65^ Useful data obtained from EPR spectra are presented as corresponding SH parameters (*g*-values, hyperfine splitting *A*, zero-field splitting *D*, *E,* etc.). Well-developed theoretical tools in combination with state-of-the-art experimental techniques allow one to obtain tiny details of a defect’s nature, surroundings, and interactions. However, an EPR study of diamond samples has its specificity dictated by the form of a diamond sample. In many cases, conventional EPR deals with bulky samples. In contrast to well-ordered, single crystal diamond, and polycrystalline diamond films, most bulky diamond samples are in polycrystalline form. Especially it concerns artificial diamonds fabricated by either static (HPHT or CVD growth) or dynamic (detonation, laser ablation) synthesis techniques in the form of micrometer- or nanometer-sized powders, a typical sample is an ensemble of small particles characterized by a distribution of individual sizes and properties of these particles. Thus, EPR spectra of most synthetic diamond samples are spatially averaged, which causes certain decreases in spectral resolution, reduction in sensitivity (due to additional line broadening), and complications in interpretation of the EPR data. One who is studying defects in various diamond samples by means of magnetic resonance (MR) must take all the aforementioned aspects into account.

Real diamond samples demonstrate a large variety of magnetic properties which descend mainly from the origin of the samples as well as any subsequent treatments performed on them. For instance, each small diamond particle obtained by micronization (milling) of bulk diamonds inherit, first of all, magnetic features of their ancestors. This means almost the entire set of paramagnetic defects and potential ferromagnetic inclusions contained in the bulk samples before micronization will be transferred into these diamond particles. Moreover, the micronization process itself creates new carbon-inherited defects within surface layers of diamond particles and may be viewed as a source of processing originated magnetic impurities. First, there should be clear discrimination between two types of defects: (1) those that are either inherent or induced in the *diamond core* or on the *surface of the diamond particle* as a result of its origin or synthesis method (e.g., metal impurity atoms from catalysts) and (2) those that are not directly induced on or within the diamond, but are instead unrelated magnetically active impurities which may stem from various processing techniques (e.g., impurity contamination from micronization processing). In both research and practical applications of diamonds, uncontrolled impurities are undesirable species that may (and have to) be removed by special purification techniques—see, for instance, Refs. 66–69. In this respect, EPR is a powerful analysis tool for assessing the content of initial magnetic impurities and the efficacy of purification procedures. Besides this aspect, magnetism due to undesirable impurities can also be assessed, but this is beyond the scope of the present review. Nonimpurity magnetism in diamonds originates from three main sources: (i) intrinsic paramagnetic defects inherited from source materials, where these defects were created in the process of diamond formation or synthesis (e.g., metallic impurities from catalysts, nitrogen); (ii) defects induced by micronization (e.g., dangling bonds along cleavage planes); and (iii) intentionally induced defects introduced to modify the properties of the initial diamond material (e.g., NV^−^ centers or other irradiation-induced defects). The latter type of defect may be subclassified into (1) defects attached to the diamond surface and subsurface interface layers by chemical modification of the surface (particularly in smaller particles with high specific surface area) and (2) defects induced by ionizing irradiation/ion implantation of raw diamond materials or resulting small particles. Each of the three major classes of magnetic defects observed in diamond is discussed below.

**Fig. 9.**
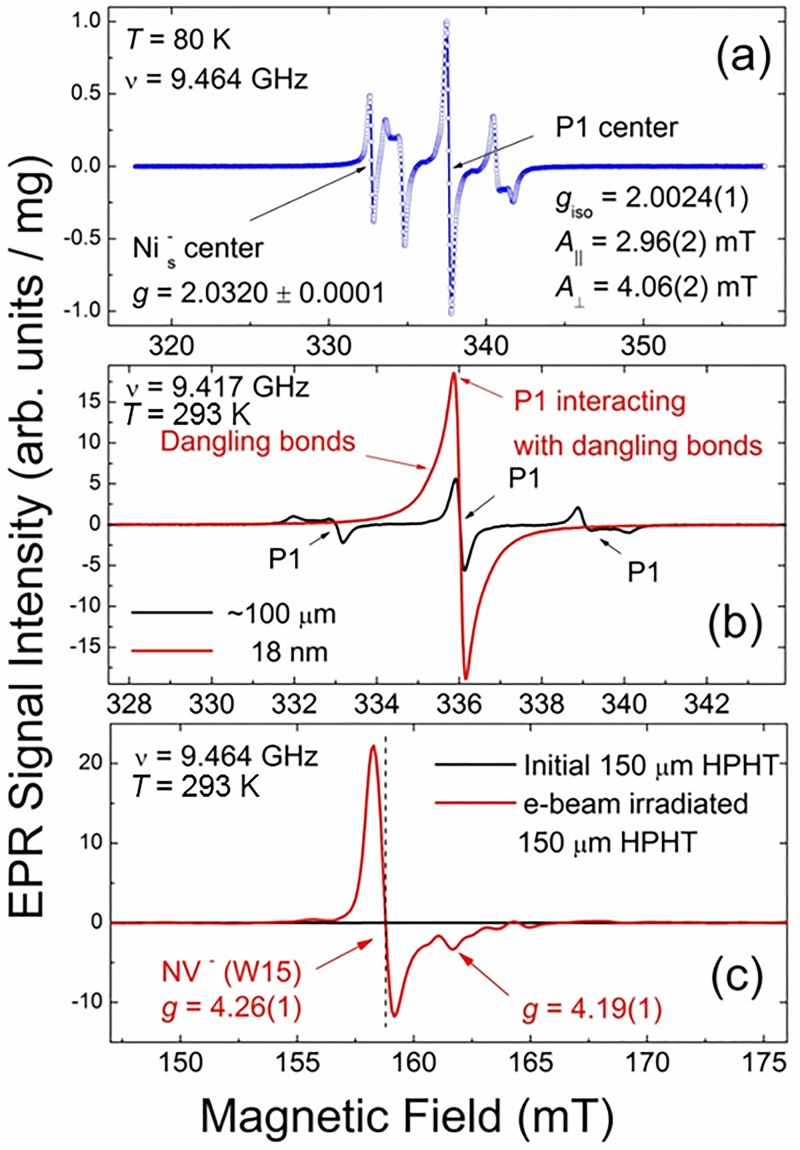
X-band EPR spectra of polycrystalline type Ib HPHT diamond samples: (a) average particle size 105–125 *μ*m, recorded at T = 80 K, ν  = 9.464 GHz; (b) average particle size ∼100 *μ*m (black trace) and 18 nm diamonds obtained by micronization of the initial sample at RT, ν  = 9.417 GHz; (c) initial (black trace) and e-beam irradiated/annealed (red trace) 150 *μ*m diamonds, half-field (region g = 4.00) signals, at RT, ν  = 9.464 GHz. Intensities of EPR spectra in (b) and (c) are normalized per unit mass.
  Defects of this type are the most abundant in bulk diamonds, both natural and synthetic. The density of P1 centers in this sample is 6.7 × 10^18^ spin/g, which corresponds well with the expected nitrogen content of type Ib diamond (∼100 ppm of nitrogen). This indicates that most of the nitrogen contained in this diamond is in the form of paramagnetic substitutional nitrogen which is, more or less, homogeneously distributed over the diamond bulk. The singlet, narrow line with *g* = 2.032 in Fig. 9(a) is attributed to substitutional nickel negatively charged defects designated as Ni_s_^−^. The density of Ni_s_^−^ centers is ∼10^17 ^spin/g. These paramagnetic defects appear during the growth of diamond crystallites from Ni-containing metal solvents and are the second (after nitrogen) most abundant defect in HPHT grown diamonds.^73^ At temperatures above 150 K (−123 °C), the EPR line of Ni_s_^−^ drastically broadens and becomes unobservable by conventional EPR.
(2) Micronization induced paramagnetic defects. Synthetic diamonds of various particle sizes (from tens of micrometers to a few nanometers) may be produced from initial micrometer-sized HPHT crystallites by micronization.^69,74^ Figure 9(b) demonstrates changes occurring in the room temperature (RT) EPR spectrum of the resulting 18 nm sample (red trace) in comparison with the spectrum of its micrometer-sized precursor (black trace).^69^ Upon decrease of the average particle size, the conventional P1 signal disappears, whereas a new, intense singlet line appears. The EPR spectrum of 18 nm nanodiamond provides a singlet signal with *g* = 2.0028 and a complicated line shape: superposition of two Lorentzian lines (broad and narrow) with indistinguishable *g*-factors. The total density of paramagnetic centers increases with decreasing average particle size: from 6.7 × 10^18^ spin/g in the initial micron-sized sample to 3.3 × 10^19^ spin/g in 18 nm ND particles.^69^ Thus, micronization creates new paramagnetic defects in smaller nanodiamond particles with *g* = 2.0028 and line width Δ*H*_pp_ = 0.93 mT, responsible for the growth of total spin density. The origin of the new defects is mechanical deformation, inducing carbon-inherited defects (mainly the uncoupled electron spins of broken bonds) located on the nanodiamond surface or within the distorted diamond subsurface lattice. The narrow signal Δ*H*_pp_ = 0.26 mT is supposedly attributed to the same P1 centers as were in the starting material, but in this sample, the P1 spectrum itself underwent drastic transformation from the characteristic hyperfine pattern to the narrow singlet Lorentzian line—see Fig. 9(b). Such a transformation of the spectrum may occur due to dipole-dipole and exchange interactions of induced spins located on the surface or within the subsurface layers with spins of P1 defects located in the core of ND particles.^69^ It is worth mentioning that the same intense singlet double component EPR signals are a characteristic feature of all nanodiamonds obtained by dynamic synthesis.^66,67^
(3) Artificially introduced paramagnetic defects. Among the variety of interesting and technologically valuable induced paramagnetic defects in diamonds, the negatively charged nitrogen-vacancy color center (NV^−^ or W15 center) with triplet electronic spin properties (*S* = 1) is the most studied. NV^−^ centers can be created in bulk diamonds containing substitutional nitrogen. Irradiation of micrometer-sized diamond crystals by a beam of high-energy (≤20 MeV) particles creates a large number of both neutral and negatively charged vacancies (V^0^ and V^−^, respectively). V^−^ defects are paramagnetic having *S* = 3/2.^75^ Subsequent annealing of the irradiated diamond at *T* ≥ 850 °C causes thermal diffusion of the vacancies and formation of NV^−^ complexes consisting of vacancies stabilized in crystalline positions adjacent to a substitutional nitrogen. EPR spectra of irradiated diamonds demonstrate, in addition to intense signals of P1, V^−^, and dangling bonds, a less intense group of satellite lines originating from conventional “allowed” (ΔM_s_ = 1) transitions between energy levels of the electronic triplet (*S* = 1) state. These lines are observed in low- and high-field regions of the main spectrum. However, the most prominent features of the EPR spectra of polycrystalline irradiated diamond samples are well observed lines in the half-field (HF) region (*g* = 4.00), attributed to so-called “forbidden” (ΔM_s_ = 2) transitions—see Fig. 9(c), red trace. The observation of these HF lines reliably indicates the appearance of irradiation-induced triplet centers. Among them, the *g* = 4.26 line is unambiguously attributed to NV^−^ centers.^76^ The spin density of NV^−^ defects in the irradiated diamond particles is found to be a function of fluence, size, and annealing temperature-dependent value (see below). Thus, the NV^−^ content in a 150 *μ*m HPHT sample irradiated with 10 MeV electrons and subsequent annealing at 800 °C was found to be 3.6 × 10^17^ spin/g or 10.8 ppm (by EPR). It is worth mentioning that in most cases, the “allowed” triplet-related lines are practically unobservable (resulting from a low content of triplet centers or masking by more intense signals due to primary defects or impurities) which significantly complicates correct EPR-assignment of the remaining spectral features to NV^−^ defects. Fortunately, the half-field “forbidden” lines remain well observable and become the primary EPR signature indicating the presence of NV^−^ centers in treated polycrystalline diamonds. Also of concern are nanodiamonds fabricated by dynamic synthesis techniques.^76^ It was found that changes in the integral intensity of the “forbidden” line agree quite well with changes in the NV^−^ content obtained by the correct procedure of integration of the entire NV^−^ related EPR spectrum. Thus, the EPR spectrum of the “forbidden” *g* = 4.26 line may be used as a reliable tool for comparative estimations of the NV^−^ content in fluorescent nanodiamonds, even in those cases when weak “allowed” lines are not (or hardly) observed.

(1) Intrinsic paramagnetic defects. Large single crystalline diamonds can be produced with low impurity concentrations and high structural perfection. However, unless specific steps are taken, nitrogen is readily incorporated into HPHT diamonds in relatively high concentrations (up to a few hundred ppm) and is present at levels of a few tens of ppm in CVD grown diamond.^70^ Nitrogen is also the major impurity in about 98% of natural diamond, and its content may reach 3000 ppm in some crystals. Hydrogen may be a significant source of defects in CVD diamond. Some metal solvents and catalysts (like nickel, cobalt, etc.) may also be incorporated in HPHT diamonds during growth. A variety of dopants such as boron, hydrogen, and silicon can be intentionally introduced into synthetic diamond. Extensive information on various types of defects in bulk diamonds may be found in the classic review article of Loubster and Wyk^71^ and the recent review of Newton.^64^ Baker and Newton^72^ focused particularly on the numerous types of nitrogen-related defects in diamonds. Figure 9(a) shows the EPR spectra of commercial, micron-sized type Ib synthetic HPHT diamonds recorded at *T* = 80 K (−193 °C). The central tripletlike pattern is observed within the broad temperature range and represents the typical spectrum of dispersed substitutional nitrogen in the polycrystalline diamond lattice—so-called P1 (or N_s_, or N^0^) centers. The uncoupled electron in this defect occupies an N-C antibonding orbital. The P1 center is neutral and has C_3v_ symmetry. The SH parameters for this singlet (*S* = 1/2) paramagnetic center are listed in the figure.

##### b. Role of e-beam fluence and annealing on radiation defects evolution and photoluminescence brightness

**Fig. 10.**
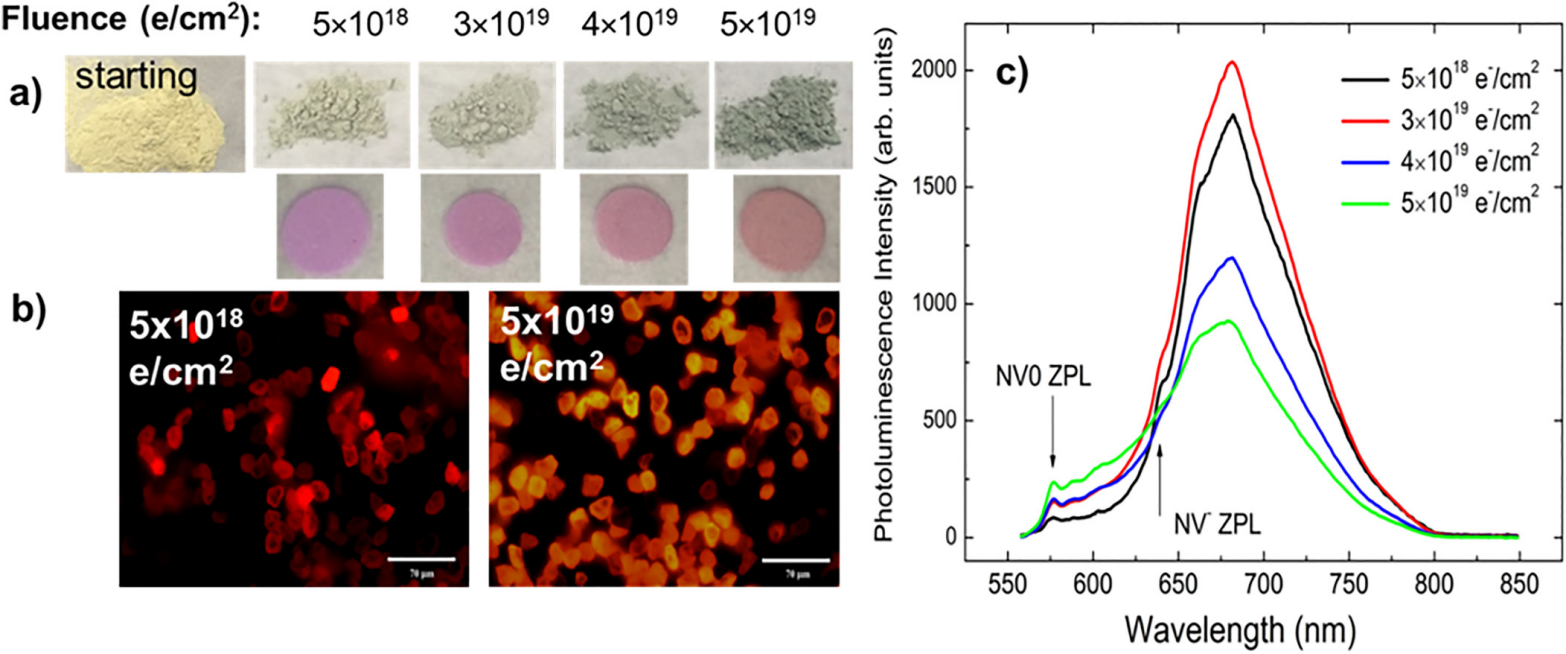
(a) A photograph of starting 15 *μ*m diamond powder (yellow) and representative samples irradiated to different fluences without (green powders) and with subsequent annealing, producing NV^−^ centers (pink powders). (b) Micrographs of fluorescent 15–20 *μ*m sized HPHT diamond particles irradiated to 5 × 10^18^ and 5 × 10^19^ e^−^/cm^2^ fluences. The micrographs are taken under identical conditions of green excitation with 40× magnification to demonstrate differences in the brightness and color of the individual particles. (c) Room temperature photoluminescence spectra of powder samples of micron-sized HPHT diamond irradiated to different fluences under 532 nm excitation.

Systematic study of the effects of e-beam fluence and annealing on the efficiency of NV^−^ formation in diamonds is within the scope of interest for both researchers and technologists. It is intuitive to think that the fluence should be as high as possible in order to achieve maximum available NV^−^ content, i.e., 25%–30% of N_s_.^77^ However, the fluence must also stay below a threshold value at which vacancy–vacancy aggregation becomes efficient (multivacancy clusters can act as electron acceptors) and below the critical density of the defects (ca. 10^22^ vacancies/cm^3^) where the onset of graphitization is observed.^78^ These considerations call for optimization of the e-beam fluence. In practice, unjustifiably high fluences also increase the treatment time and the production cost dramatically. Thus, in order to address these issues, the efficiency of NV^−^ center formation in 15–20 *μ*m sized HPHT particles has been studied by EPR as a function of the incremental e-beam fluence followed by vacuum annealing at 850 °C.^60^ The fluences in this series were (in e^−^/cm^2^): 5 × 10^18^, 1 × 10^19^, 1.7 × 10^19^, 3 × 10^19^, 4 × 10^19^, and 5 × 10^19^. Figure 10(a) illustrates powder irradiated to different fluences without and with annealing. After irradiation, the initially yellow powder (due to light absorption by nitrogen) acquires a green color, which changes from pale green to saturated green as the fluence increases. The main reason for the radiation-induced green color is light absorption of GR1 centers (neutral vacancies with a ZPL at 741 nm) which occurs in the yellow/orange part of the spectrum. A vacancy is an electron acceptor, and in a sample with a high concentration of substitutional N (Ns), vacancies become negatively charged (so-called ND1 centers). Besides ND1 and GR1, divacancies and vacancy clusters are also expected.^79,80^ A small amount of brown coloration appears at irradiation fluences of 3 × 10^19^ e^−^/cm^2^ and higher is an indication that vacancy clusters have started to form. Figures 10(b) and 10(c) also illustrate that the brightness of the sample irradiated to the lowest fluence (5 × 10^18^ e^−^/cm^2^) is higher than that of the sample irradiated to the highest fluence (5 × 10^19^ e^−^/cm^2^). While the high brightness of the sample irradiated to 5 × 10^19^ e^−^/cm^2^ is also evident from the micrograph, it can be attributed to significant luminescent contribution from NV^0^ centers.

**Fig. 11.**
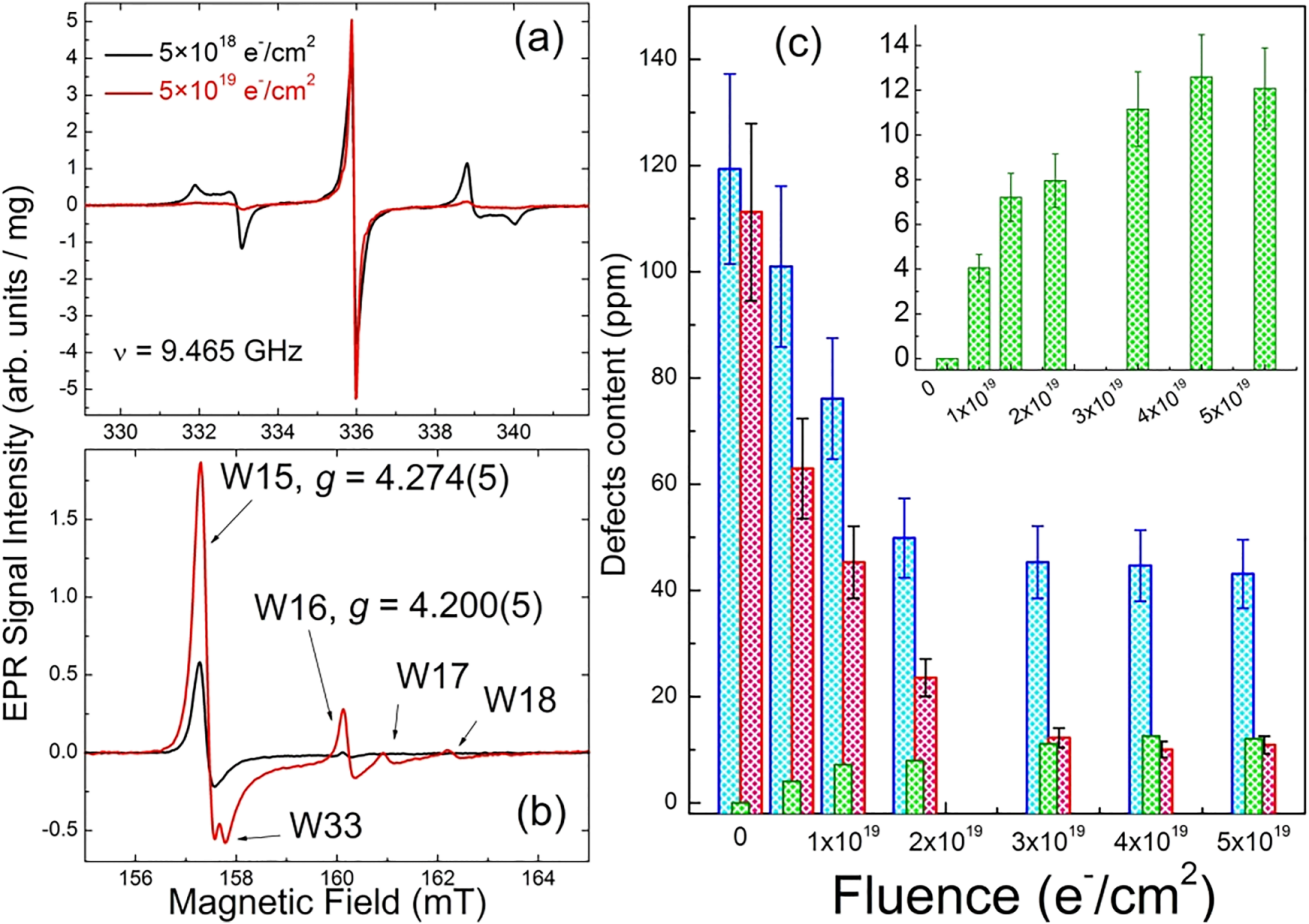
(a) and (b) RT X-band EPR spectra of the 15–20 *μ*m particle size polycrystalline type Ib HPHT diamond samples having undergone e-beam irradiation and 850 °C vacuum annealing, black traces—fluence 5 × 10^18^ e^−^/cm^2^, red traces—fluence 5 × 10^19^ e^−^/cm^2^, ν  = 9.465 GHz: (a) g = 2.00 region, primary *S* = 1/2, 3/2 defects, (b) half-field *g* = 4.00 region, triplet *S* = 1 defects; intensities of EPR spectra in (a) and (b) are normalized per unit mass; (c) dependence of the content of some paramagnetic defects on e-beam fluence: blue columns—total primary defects, red columns—P1 centers, green columns—NV^−^ centers. Inset—zoom for NV^−^ centers.

Figure 11 summarizes results of the EPR study done on this series. The obtained EPR data provide a clear demonstration that intense electron beam irradiation causes a significant fluence-dependent reduction of the content of P1 centers inherent in as-manufactured HPHT diamonds—see Figs. 11(a) and 11(c). This P1 center reduction is accompanied by the appearance of a new structureless EPR line with about the same (within the experimental error) *g*-factor attributed to negative vacancies V^−^.^2^ The capture of the electrons from the substitutional nitrogen sites by these vacancies leads to the formation of positively charged, nonparamagnetic P1^+^ centers. The same vacancies are expected to become mobile at annealing temperatures and, therefore, are responsible for the formation and further evolution of various paramagnetic species with *S* = 1. *EasySpin* simulation^81^ of the half-field EPR polycrystalline patterns in Fig. 11(b) allowed for reliable assignment of the “forbidden” lines to various triplet centers previously observed in irradiated diamond samples. Thus, in addition to the major NV^−^ (W15) center, W16–W18 and W33 triplet centers were identified. At low fluences, mainly W15 triplet centers are formed [Fig. 11(b), black trace]. An increase in the fluence causes progressive growth of the W15 [Fig. 11(c), inset], W16–W18, and W33 triplet centers—see Fig. 11(b), red trace. At the highest e-beam fluence of 5 × 10^19^ e^−^/cm^2^, the relative abundances of the triplet centers were found to be 1(W15):0.18(W16):0.03(W17):0.02(W18):0.02(W33).^60^

**Fig. 12.**
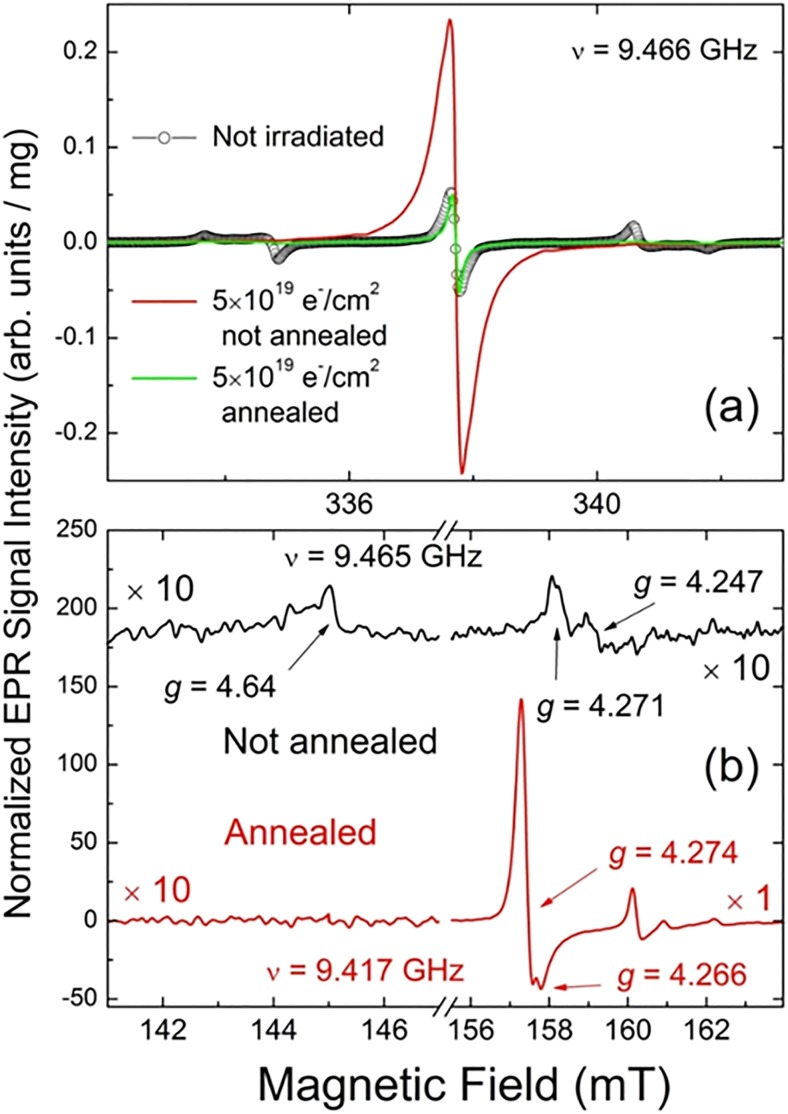
Room temperature X-band EPR spectra of the 15–20 *μ*m particle size polycrystalline type Ib HPHT diamond: (a) g = 2.00 region, primary *S* = 1/2, 3/2 defects, ν = 9.466 GHz. Black trace—initial nonirradiated sample, red trace—5 × 10^19^ e^−^/cm^2^ irradiated sample, not annealed, green trace—5 × 10^19^ e^−^/cm^2^ irradiated sample, vacuum annealed at 850 °C; (b) half-field *g* = 4.00 region, triplet *S* = 1 defects. Black trace—5 × 10^19^ e^−^/cm^2^ irradiated sample, not annealed, ν = 9.465 GHz, red trace—5 × 10^19^ e^−^/cm^2^ irradiated sample, vacuum annealed at 850 °C, ν = 9.417 GHz. Intensities of EPR spectra in (a) and (b) are normalized per unit mass; spectra in (b) are vertically shifted for better presentation. Intensities of the spectra of the irradiated unannealed sample in (b) as well as the lower field part of the irradiated annealed sample are plotted with the multiplication coefficient of 10 Arrows in (b) point out the positions and g-factors of NV^−^ (W15), W33 and R1/R2 “forbidden” lines.

The role of annealing in the formation of triplet centers in e-beam irradiated diamonds is no less interesting than the study of fluence dependence. The same initial 15–20 *μ*m HPHT diamond sample as above has been studied in its irradiated unannealed and annealed forms.^61^ Figure 12 demonstrates the main features observed in the system of paramagnetic defects that appear in the diamond structure as a result of e-beam irradiation and following annealing of the irradiated sample in vacuum at 850 °C. The initial unirradiated sample [Fig. 12(a), black trace] contains mainly P1 canters (∼110 ppm) and dangling bonds ∼10 ppm with no detectable traces of NV^−^ and other triplet defects typical for irradiated diamonds.

As is shown in the plot, the EPR spectra of the same sample irradiated to 5 × 10^19^ e^−^/cm^2^ fluence and unannealed clearly demonstrate a ten times drop in the P1 content accompanied by the rise of an intense singlet structureless signal with *g* ∼2.003 attributed to V^−^ defects—see red trace in Fig. 12(a). The V^−^ content in the unannealed sample is estimated to be ∼550 ppm. Half-field spectra of the unannealed sample indicate the presence of a small amount of NV^−^ (∼0.2 ppm), W33 (<0.2 ppm), and R1/R2 (∼0.5 ppm) triplet defects [Fig. 12(b)]. The R1/R2 centers have been attributed to interstitial defects.^82^ Vacuum annealing of the irradiated sample at 850 °C dramatically changes the abundance of the paramagnetic defects in the diamond particles. The green trace in Fig. 12(a) indicates a collapse of the V^−^ related signal, whereas the weak P1 signal remains the same. The remaining V^−^ signal in the annealed sample corresponds to only ∼20 ppm. On the other hand, in the half-field region, the 20-fold peak-to-peak intensity rise and a narrowing of the NV^−^ signal as well as the appearance of the well detectable W16-W18 and W33 signals are observed—see the higher field part of the red trace in Fig. 12(b). The content of NV^−^ centers in the irradiated and annealed sample is found to be 12 ppm. At the same time, the R1/R2 interstitial triplets signals observed in the unannealed samples disappear upon annealing—compare the lower field parts of the spectra in Fig. 12(b). Thus, it may be concluded that intense electron irradiation creates a huge amount of weakly bound negatively charged lattice defects (vacancies). This process is accompanied by charging substitutional nitrogen (P1 centers), thus reducing their content. Some of these vacancies happen upon P1 centers, causing formation of rare NV^−^ and W33 centers. Intense e-beam irradiation also creates interstitial triplet defects of R1/R2 type. Conventional vacuum annealing accelerates the mobility of V^−^ which causes intensive formation of NV^−^ and other triplet defects and quenching of interstitial triplet defects. In this way, the content of fluorescent NV^−^ triplet centers increases about 60 times, reaching quite a high value of 12 ppm.

Thus, an important conclusion was made that the photoluminescence intensity initially increases with increasing NV^−^ center density, but then starts to decrease (at ∼8 ppm of NV^−^ centers for 15–20 *μ*m particles). While samples irradiated to the highest fluence (5 × 10^19^ e^−^/cm^2^) contained the highest density of NV^−^ centers (∼12 ppm), their photoluminescence intensity was below that for the sample irradiated to the lowest fluence (5 × 10^18^ e^−^/cm^2^) and lowest NV^−^ density (∼4 ppm) due to possibly self-quenching of the NV^−^ centers and formation of accompanying defects.

##### c. Dependence of NV centers formation on diamond particle sizes

**Fig. 13.**
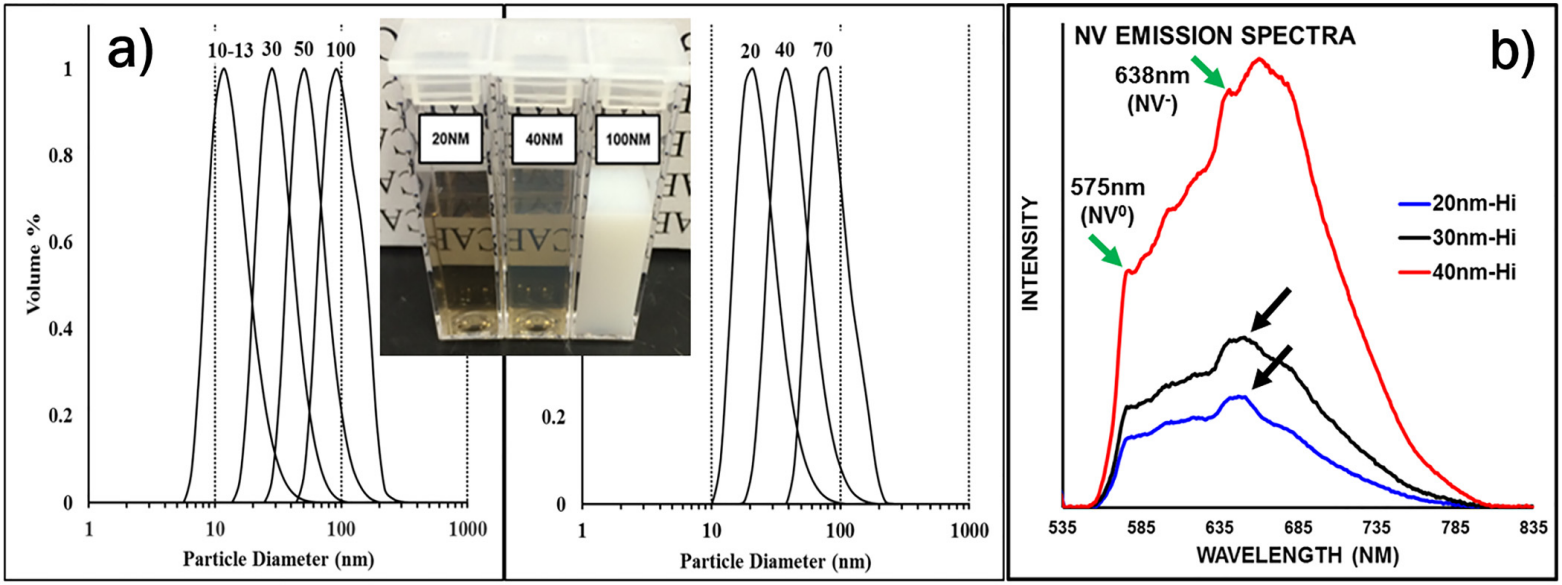
(a) Volumetric particle size distributions of smallest fractions of FND after milling as measured using DLS. The inset photo illustrates suspensions of 20, 40, and 100 nm fluorescent nanodiamond particles in deionized water. Concentrations are approximately 1 mg/ml. (b) Emission spectra of 20, 30, and 40 nm particle suspensions in deionized water at approximately 1 mg/ml (0.1% w/v) concentration. Excitation by 45 mW 532 nm CW laser. Spectra collected with an Ocean Optics HR2000 USB spectrometer with 500 msec integration time. Black arrows denote water Raman peak at ∼650 nm. Reprinted with permission from O. Shenderova *et al*., Proc. SPIE **10118**, 1011803 (2017). Copyright 2017, International Society for Optics and Photonics.

Another aspect of fluorescent diamond manufacturing is optimization of the NV^−^ content in fluorescent diamond samples having predefined particle sizes within the range from tens of micrometers to single digit nanometers. Much higher NV density can be achieved by irradiation and annealing of micron-sized particles rather than submicron particles. As such, techniques have been developed for milling micron-sized diamond using a planetary ball milling apparatus.^32^ Figure 13(a) illustrates the particle size distribution of the smallest fractions of fluorescent nanodiamonds after milling of 15–20 *μ*m particles and subsequent purification and fractionation by centrifugation. Nanodiamonds scatter light strongly, which can be useful for both directing and attenuating light. Visually, solutions of large nanodiamond particles appear milky-white down to approximately 50–70 nm, where the solution adopts a transparent brown/amber color [Fig. 13(a), inset]. As a part of material certificates of analysis for commercial products, volumetric particle size distributions are provided along with Z-average size (intensity based). It should be noted that the often-reported number-based particle size distribution from dynamic light scattering (DLS) is misleading as DLS is not a suitable technique for generating number-based distributions. Number distributions require direct image analysis from some form of optical or electron microscopy and often lead to erroneous under-representation of the particle sizes. Figure 13(b) illustrates photoluminescence spectra for 20, 30, and 40 nm particles. The brightness of the particles depends on the particle size. The larger the particle, the higher the brightness due to the larger number of color centers that can be accommodated by larger particle volumes.

**Fig. 14.**
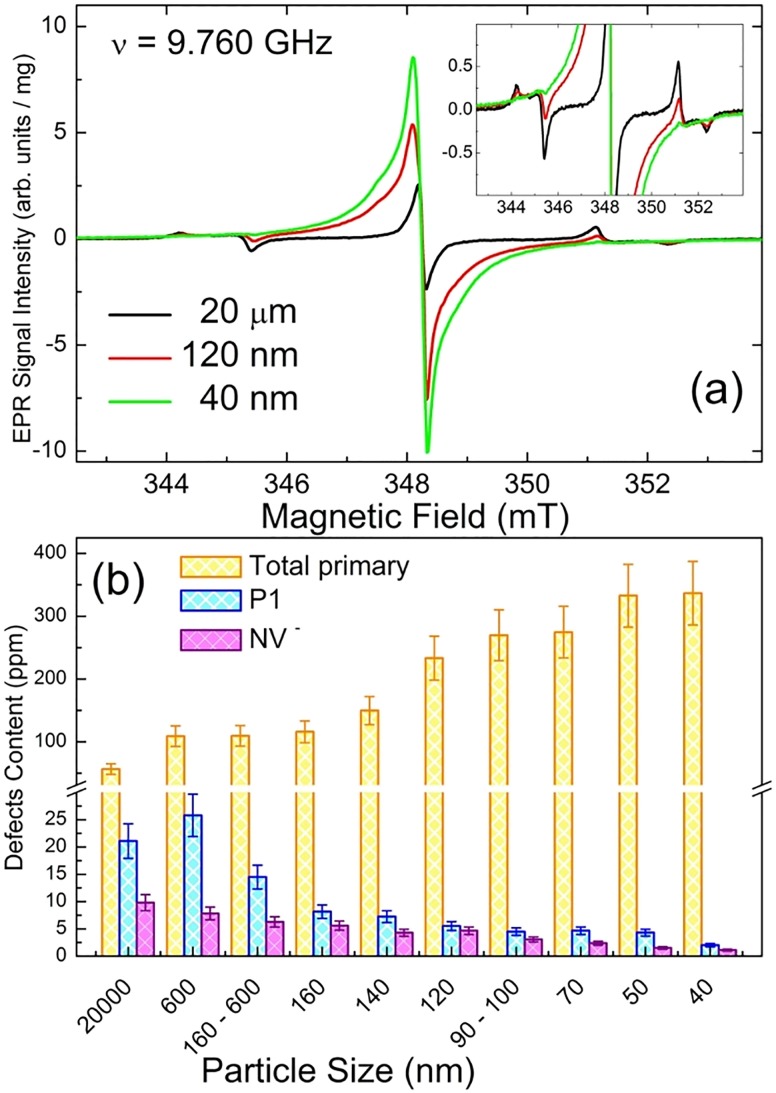
(a) Room temperature X-band EPR spectra of primary defects (*g* = 2.00 region) recorded for a series of 3 × 10^19^ e^−^/cm^2^ irradiated and annealed polycrystalline type Ib HPHT diamond samples as function of average particle size: black trace—20 *μ*m, red trace—120 nm, green trace—40 nm, ν = 9.760 GHz, intensities of EPR spectra are normalized per unit mass, inset shows vertical zoom for the satellite P1 hyperfine signals; (b) dependence of the content of some paramagnetic defects on average particle size: yellow columns—total primary (*S* = 1/2, 3/2) defects, cyan columns—P1 centers, magenta columns—NV^−^ centers.

It was recently shown that micronization of the initial irradiated and annealed micrometer-sized fluorescent diamond particles to nanometer-sized particles causes a consequent reduction of the EPR detected NV^−^ content.^68^ Figure 14 summarizes results of EPR analysis of the effect of micronization on a series of 3 × 10^19^ e^−^/cm^2^ irradiated and annealed polycrystalline type Ib HPHT diamond samples obtained by milling of the initial 15–20 *μ*m fluorescent diamond particles and subsequent separation by sizes. The EPR spectrum of the initial sample recorded in the region of *g* = 2.00 indicates a certain content of primary defects: mostly P1 centers and V^−^ as well as a small amount of dangling bonds—see black trace in Figs. 14(a) and 14(b). The half-field EPR spectrum shows a well pronounced *g* = 4.25(1) signal—characteristic of NV^−^ centers (not shown). The NV^−^ content in the initial sample is found to be ∼10 ppm by EPR. Reduction of the average particle sizes to the hundreds of nanometers range causes a well pronounced reduction of the isolated P1 centers content (responsible for the characteristic hyperfine structure signals) and growth of the central structureless line clearly consisting of at least two components—broad and narrow ones [red trace in Fig. 14(a)]. On further decrease of particle size, the isolated P1 lines become practically unobservable, whereas the central two-component signal continues to grow [green trace in Fig. 14(a)]. Following Panich *et al.*,^69^ the broad component of the central line is attributed to dangling bonds whose content continuously increases with decreasing particle size, and the narrow signal is mostly due to P1 centers interacting with dangling bonds. At the same time, the content of EPR detected NV^−^ centers consistently goes down with smaller particle sizes [Fig. 14(b)]: for the 40 nm sample, the NV^−^ content remains only 1 ppm. Thus, the study above justifies the conclusion drawn by Shames *et al.*:^68^ micronization of relatively large micrometer-sized particles to nanometer-sized ones is accompanied by a significant reduction of the NV^−^ content.

**Fig. 15.**
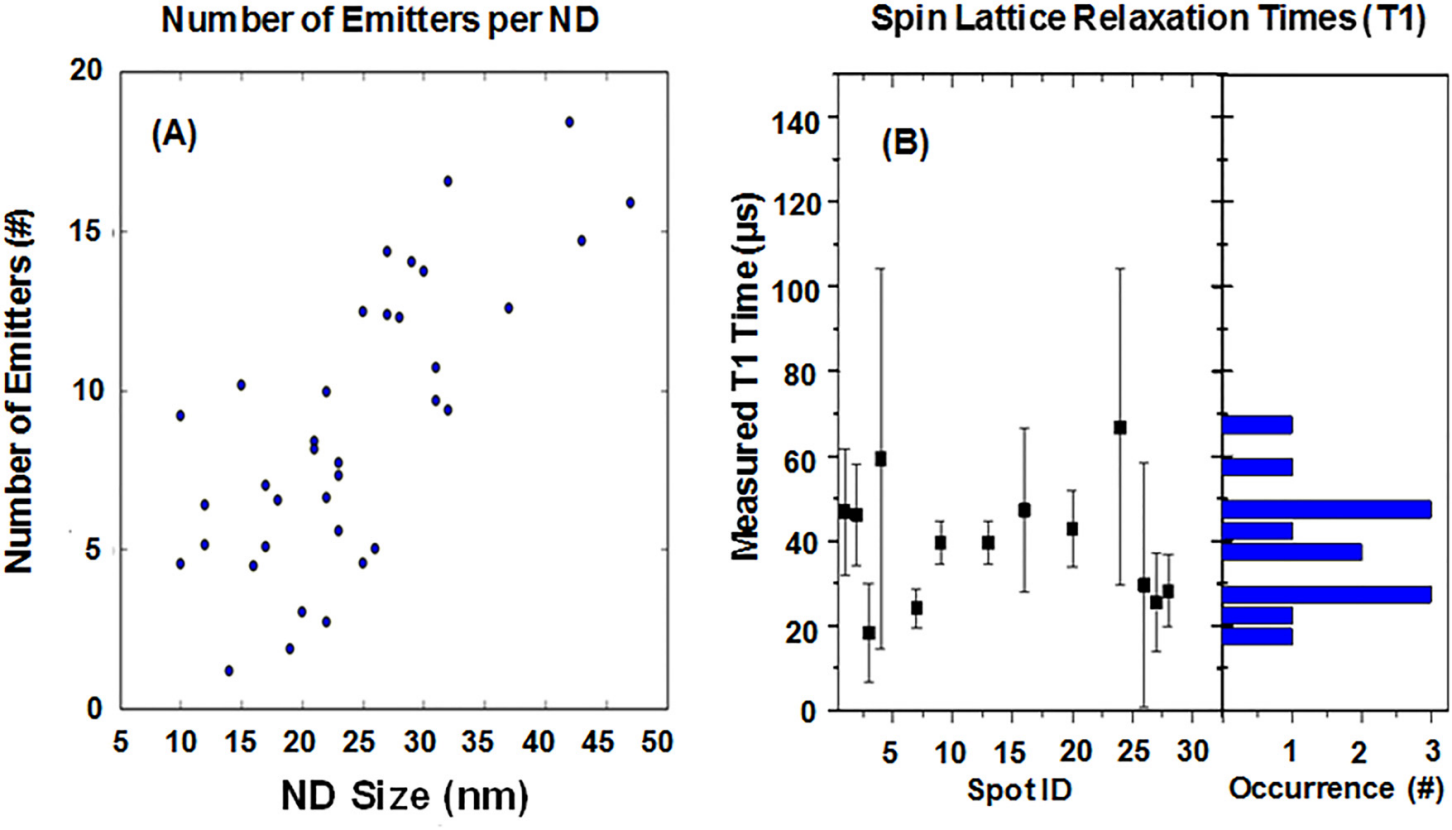
(a) Trend of number of NV^−^ emitters per particle vs particle size. While there is a reasonable amount of statistical error inherent in these data due to the small sample sizes, a general trend of decreasing number of NV^−^ centers per particle with decreasing particle size is observed. (b) Spin-lattice (T1) relaxation times in 34 different particles approximately 20 nm in size dispersed over a glass substrate. T1 times are on the order of 30–40 *μ*s, which are still significantly less than values reported for bulk diamond, which can exhibit T1 times on the order of the millisecond timescale (Ref. 86). Reprinted with permission from O. Shenderova *et al*., Proc. SPIE **10118**, 1011803 (2017). Copyright 2017, International Society for Optics and Photonics.

While diamond particles with sizes as small as 10–20 nm containing NV centers have become commercially available,^32^ there are a number of fundamental material challenges that still need to be solved, and, in general, these have been previously stated in the literature as well.^12,32^ Briefly, key opportunities for development revolve around improving the quality of NV centers in particulate diamond, particularly at sizes <30 nm. While a number of groups,^32,83–85^ including ours,^32^ have attempted to improve the quality of these “quantum” fluorescent diamond particles, and it is generally accepted that the quality needed has not yet been achieved. We report some of our more recent single particle analysis of 10–40 nm particles in Table II and Fig. 15.

**Table II.** Single particle AFM confocal analysis of several FND samples ranging in size from 10 to 40 nm. The analysis technique was previously reported (Ref. 32), and was performed on a sample of a few hundred particles per size. Measurements courtesy of T. Oeckinghaus (U. Stuttgart).

Particle size (nm)	# NV^−^ emitters/particle	Percent of fluorescent particles in sample
10	1–2	6
20	1–2	28
30	5–6	57
40	1–4	74
40^a^	12–14	70

^a^ From higher electron irradiation batch.

The process of crushing bulk diamond leaves a population of some particles that do not contain color centers (Table II). Size (height) distributions of the samples as measured by atomic force microscopy (AFM) are smaller than those obtained by dynamic light scattering measurements, suggesting that the particles might have a platelike shape. The presence of platelike particles was observed in a high resolution transmission electron microscope (HRTEM).^32^

##### d. Role of postsynthesis purification

**Fig. 16.**
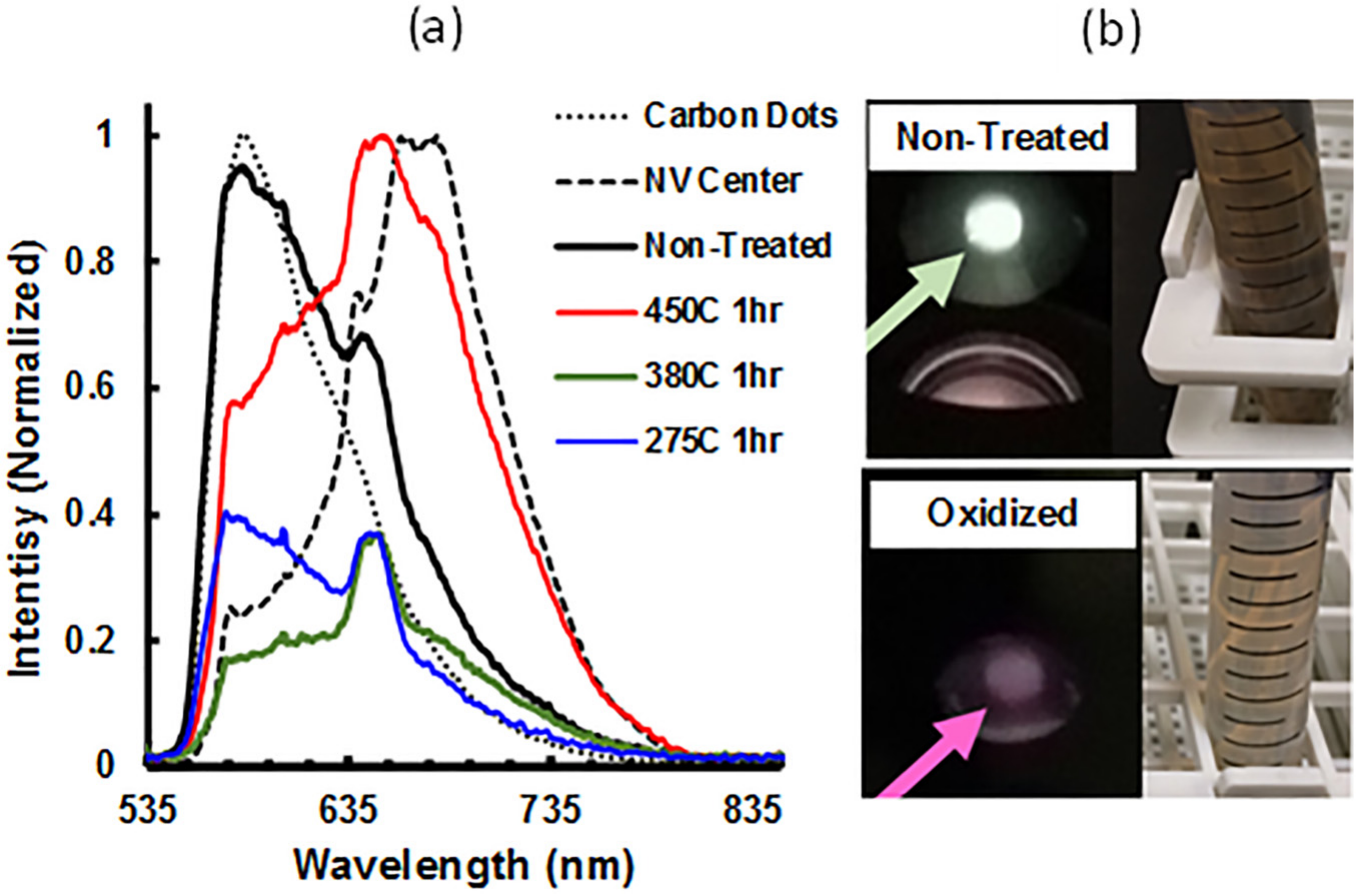
(a) Fluorescence spectra of milled HPHT diamond solutions of approximately 30–40 nm particles following a series of air oxidation treatments. Excitation was a 532 nm CW laser with 5000 msec integration time with an Ocean Optics HR2000 USB Spectrometer. The water Raman peak is observed at approximately 650 nm in all samples. The sharp edge near 560 nm is associated with the emission filter. Typical spectra associated with carbon dots and NV centers under similar 532 nm excitation are overlaid for comparison. (b) Droplets of nonoxidized and oxidized milled fluorescent diamond particles under UV excitation (4′6,-diamindino-2-pyenylindole) with corresponding bulk suspensions of each material, demonstrating the higher optical absorption of the nonoxidized sample with respect to the oxidized sample.

The removal of amorphous carbon following milling plays a significant role in the ultimate brightness of the FDPs. However, the method of purification can also have a significant impact on the yield of diamond recovered as well, which directly impacts the cost of the material and is an important factor in the evaluation process efficiency: a purification scheme which provides bright particles, but simultaneously causes significant mass losses is not optimal. An example of how postsynthesis processing can have large effects relates to fluorescence from amorphous carbon species (e.g., carbon dots), which has been demonstrated in detonation nanodiamond (DND).^87–90^ Our group has recently observed similar fluorescence in milled HPHT fluorescent diamond. This amorphous carbon related fluorescence is characterized by a fluorescence band around 580–590 nm, which is distinct from fluorescence associated with nitrogen-vacancy (NV) centers, which have a fluorescence peak at roughly 680 nm (Fig. 16).

The data presented in Fig. 16 show the peak associated with amorphous carbon fluorescence in HPHT is diminished following even modest air oxidation at 275 °C. Additionally, comparison of the fluorescence of the amorphous carbon with the fluorescence of isolated carbon dots demonstrates that the origins of this fluorescence in the milled diamond are similar. The fluorescence becomes red-shifted following higher temperature oxidation treatments, which correlates to a red-shift in the fluorescent due to lessened contribution from amorphous carbon and greater contribution from NV centers within the diamond particles. The exact nature of this amorphous carbon fluorescence is not understood. It is hypothesized that this fluorescence may originate from amorphous carbon on the surfaces of the diamond particles, similar to what was previously observed with detonation nanodiamond.^87–90^ Alternatively, this fluorescence may be the result of free-standing amorphous carbon particles, resulting from very small fragments of cleaved diamond particulate, which are completely amorphized during milling. A combination of both such states of amorphous carbon may also be present. The very low temperature stability of the amorphous fluorescent species suggests that a large proportion may be free amorphous carbon as opposed to diamond surface amorphous carbon. It has been shown in the literature that oxidation of both HPHT diamond and DND occurs at higher temperatures (400–500 °C),^91,92^ so the apparent dramatic change in the characteristic photoluminescence after only 1 h at 275 °C is peculiar and suggests the presence of a less thermally stable fluorescence species.

**Fig. 17.**
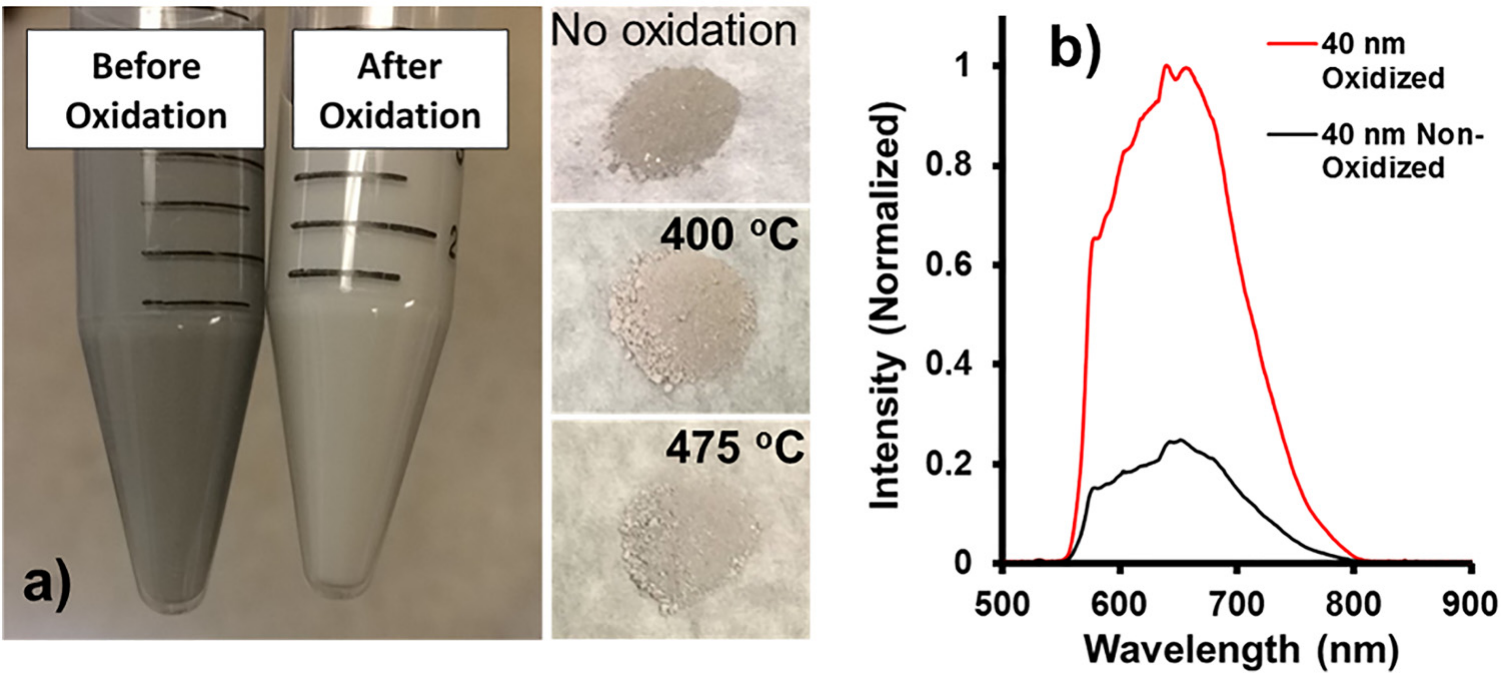
(a) Transition of color of suspensions and dry powder of milled fluorescent nanodiamonds following oxidation, indicating removal of amorphous, optically dark phase. (b) Brightness enhancement of 40 nm FNDs following air oxidation after milling.

The increase in the photoluminescence with additional air treatment may be associated with (1) the removal of surface amorphous carbon from diamond particles, (2) an increase in the overall population of fluorescent particles by complete oxidation of less stable diamond particles, which may have been damaged significantly during milling, (3) or a combination of both factors. A significant lightening of the color of diamond powder and suspensions [Fig. 17(a)] is observed following oxidation. An approximate 5× brightness enhancement is observed following oxidation. Additionally, a more pronounced ZPL associated with the NV^−^ center at approximately 638 nm is observed following oxidation [Fig. 17(b)]. Likewise, excitation with UV radiation confirms the red-shifted fluorescent spectra [Fig. 16(b)].

##### e. Role of initial nitrogen content on photoluminescence intensity

In conjunction with the aforementioned issues of e-beam fluence, annealing, and particle size, the optimization of brightness in NV^−^ based fluorescent diamonds must take into account the initial nitrogen content. On the one hand, the creation of NV^−^ centers demands the presence of a certain density of interstitial nitrogen (P1 centers), which is a precursor of NV^−^ centers. On the other hand, increasing the substitutional nitrogen content in the diamond crystal also causes crystal imperfectness due to formation of undesirable pairs, such as B-type (4N-V complexes), H3 centers (N-V-N in a neutral charge state), clustered nitrogen (tens of nitrogen atoms accumulated within areas of ∼3–4 nm sizes), etc.^1^ Moreover, simply increasing the substitutional nitrogen density might not lead to desirable increases in NV^−^ emission. Indeed, one opposing process that might accompany any increase in NV^−^ density is a concomitant increase in other fluorescence quenching processes. As a rule, defects located a few tens of nanometers from the emitting center (NV^−^ in this case), effectively quench PL by several mechanisms. Quenching mechanisms could include capture of photoexcited electrons by different defects in the crystal lattice and other nonradiative channels of energy dissipation such as Foster resonant energy transfer.^93^ A combined EPR and PL study of the initial nitrogen content has focused on answering some of the problematic issues above.^94^ The comparative study was done on two 5–20 *μ*m sized HPHT diamond powders grown with different nitrogen content—samples LN (low nitrogen) and HN (high nitrogen), and then irradiated to the same 7 × 10^18^ e^−^/cm^2^ e-beam fluence and annealed at 850–900 °C for several hours—samples LNE (low nitrogen electron irradiated and annealed) and HNE (high nitrogen electron irradiated and annealed), correspondingly. EPR spectra of the initial unirradiated LN and HN samples recorded within the *g* = 2.00 region (not shown) demonstrate a complicated signal consisting of a superimposed triplet hyperfine pattern, characteristic of the isolated P1 centers, and a broad singlet Lorentzian-shaped line with about the same *g*-factor, attributed to clustered P1 centers.^94^ In the spectrum of the HN sample, the singlet line predominates. Total content of *S* = 1/2 spins in the LN and HN samples was found to be 130 and 580 ppm, respectively. Among them, the content of isolated substitutional nitrogen (P1 centers) in both LN and HN samples was found to be about the same ∼110 ppm.

**Fig. 18.**
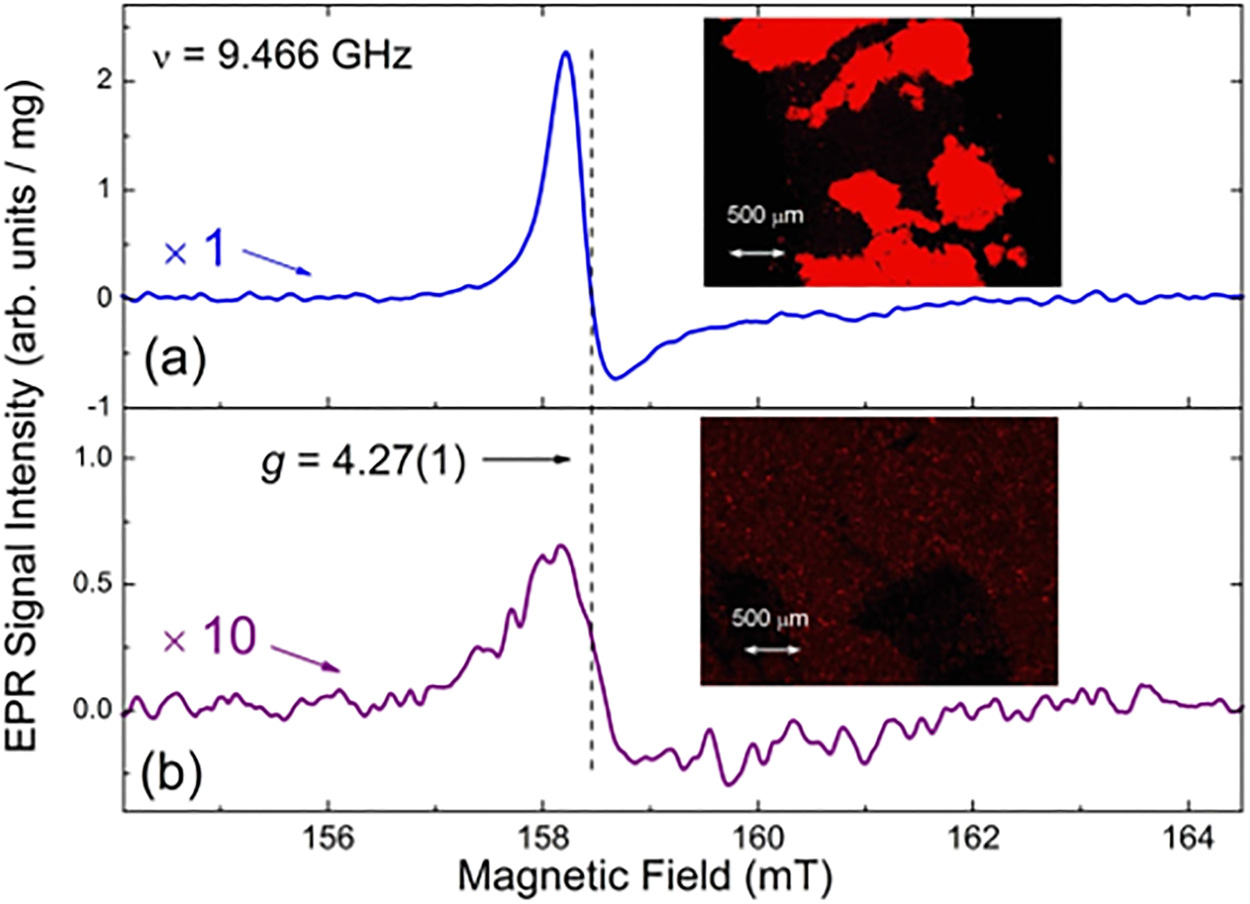
Half-filed RT X-band EPR spectra of NV^−^ defects (*g* = 4.2 region) recorded for 7 × 10^18^ e^−^/cm^2^ irradiated and annealed samples LNE (a) and HNE (b), ν = 9.466 GHz. Vertical line points out position of the characteristic “forbidden” NV^−^ line. Insets in (a) and (b) represent optical fluorescent images of samples LN irradiated and annealed and HN irradiated and annealed samples, respectively. See Ref. 94 for more information. Adapted with permission from Alexander I. Shames, Vladimir Yu. Osipov, Kirill V. Bogdanov, Alexander V. Baranov, Marianna V. Zhukovskaya, Adam Dalis, Suresh S. Vagarali, and Arfaan Rampersaud, J. Phys. Chem. C **121**, 5232 (2017). Copyright 2017, American Chemical Society.

Figure 18 represents half-field EPR spectra for irradiated/annealed LNE [Fig. 18(a)] and HNE [Fig. 18(b)] samples together with the corresponding optical images obtained using a fluorescent microscope. The peak-to-peak intensities of the “forbidden” lines in Fig. 18 are obviously different, but the “forbidden” line in HNE was found to be about three times broader than that in LNE. As a result, the NV^−^ content in LNE and HNE samples was found to be unexpectedly close: 3.4 and 4 ppm, respectively. It seems that the excessive clustered nitrogen does not participate in the formation of NV^−^ centers. However, the most interesting effect is observed in the fluorescence images: diamond particles of the LNE sample, having slightly less NV^−^ content than HNE, provide a much brighter image—see insets in Fig. 18. The intensity of the PL emission from NV^−^ centers in the LNE sample is found to be four times stronger in comparison with HNE. It may be concluded that the NV^−^ PL intensity is controlled not solely by the content of NV^−^ centers but, to a large extent, by the presence of nitrogen-related crystal defects. Increasing the nitrogen content during HPHT synthesis correspondingly increases this structural imperfection and, thus, is responsible for the appearance of the additional nonradiative recombination channel via the defects contributing to greater PL quenching.

### C. Production of multicolor diamond particles

**Fig. 19.**
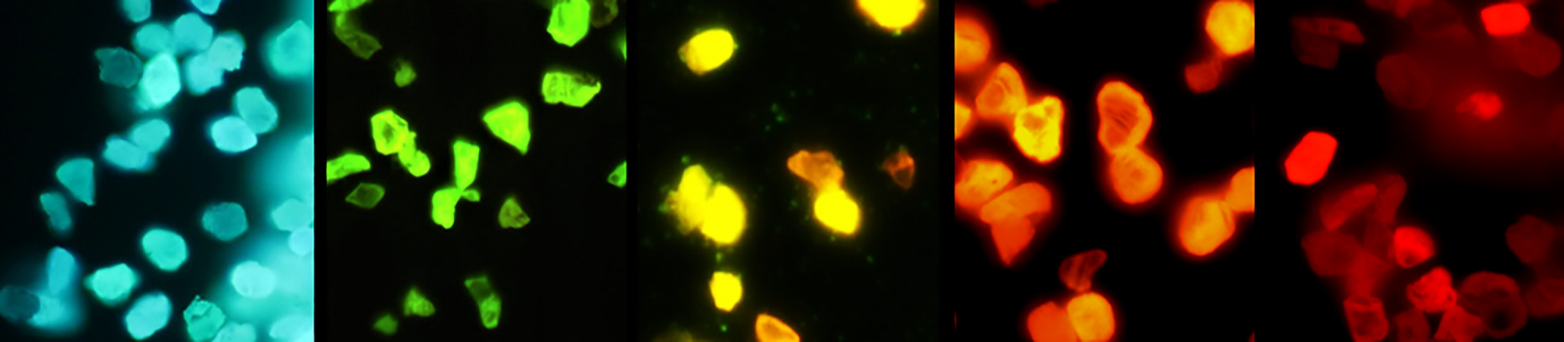
Formation of one-, two-, and three-atom nitrogen complexes with vacancies in electron irradiated type Ib synthetic diamond, using a method of rapid thermal annealing providing vibrant luminescence in the red, green, and blue spectral ranges, correspondingly.

Assemblies of lattice vacancies and substitutional nitrogen make up the majority of color centers in diamond, forming three primary variants [Fig. 1(b)]: (i) a single nitrogen (N) atom and a vacancy (V) (NV center), responsible for red/NIR fluorescence, (ii) two nitrogen atoms adjacent to a vacancy (H3 center), responsible for green fluorescence; and (iii) three nitrogen atoms surrounding a vacancy (N3 center), responsible for blue fluorescence. Commercial production is mostly centered around FDPs with red/NIR emission based on NV centers.^31–33,46^ NV centers are formed in type Ib synthetic diamond particulate, which contain approximately 100 ppm of single substitutional nitrogen, via irradiation with high-energy particles and subsequent thermal annealing (Sec. II B). Production of FDPs containing H3 centers requires the presence of nitrogen pairs within the lattice (so-called A centers). Although natural diamonds contain A centers up to 2000–3000 ppm and have been used for production of green FDP,^95^ natural diamond is inherently a poor starting material with low fluorescence brightness due to unpredictable and uncontrolled nitrogen content and an excess of A centers which quench the fluorescence of H3 centers.^1^ Blue FDPs have not been produced by this method due to the lack of a starting material containing an appreciable amount of nitrogen triplets. While A centers and H3 centers can be formed in bulk synthetic type Ib diamond via extended (hours) annealing at high temperature (exceeding 1500 °C),^96^ such annealing times and temperatures would cause complete graphitization of particulate diamond. Recently, a rapid thermal annealing technology had been developed for production of green and blue fluorescent diamond particles from synthetic diamond powder by controlled formation of H3 and N3 assemblies (Fig. 19) by varying annealing temperature in the 1000–2000 °C temperature range.^97^ Rapid heating creates conditions when high concentrations of mobile vacancies, interstitials, and nitrogen atoms coexist, providing favorable conditions for simultaneous diffusion of nitrogen atoms and vacancies and the formation of H3 centers and, at higher temperatures, N3 centers.

**Fig. 20.**
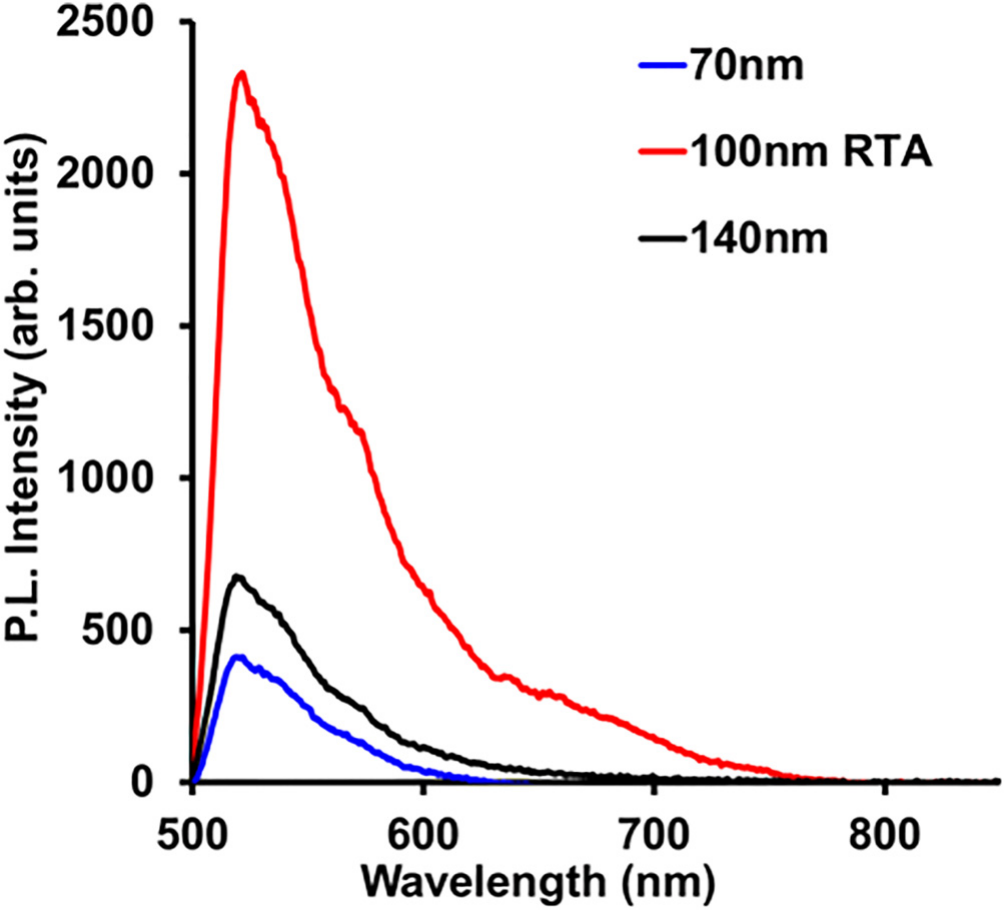
Fluorescent spectra of FND samples containing H3 centers. Samples 70 nm (blue) and 140 nm (black) are produced from natural type Ia nanodiamond particles irradiated by 3 MeV electrons (70 nm particles) or He ions (Ref. 46) followed by traditional annealing (800−900 °C, 1–2 h). The 100 nm sample (red) is produced from type Ib synthetic diamond by irradiation with 3 MeV electrons followed by rapid thermal annealing at 1900 °C for 1 min. Excitation is 465 nm (Ref. 97).

Remarkably, 100 nm irradiated type Ib diamond powder withstood rapid thermal annealing treatment at 1800 °C for 3 min, and particles with bright green luminescence were obtained, exceeding the brightness of traditionally produced green luminescent particles from natural diamond by approximately a factor of five (Fig. 20).

It is expected that multicolor particulate diamond will find broad application in industrial fields such as photonics, forensic tagging, anticounterfeiting, and fluorescent art paints. They can possibly complement quantum dots (QDs) in displays providing unprecedented durability and photostability. Multicolor fluorescent diamond particles can potentially be used for multiplexed fluorescence bioimaging, a feature which was previously not viable with FDPs due to the lack of available colors beyond just red and green. This advancement, paired with their well-established biocompatibility and outstanding photostability, makes FDPs considerably more competitive against currently existing QDs and organic dyes. Two-photon excitation between 800 and 1080 nm paired with multiplex detection is a possibility with these particles as well.

## III. APPLICATIONS OF FLUORESCENT DIAMOND PARTICLES

Transition of a novel nanomaterial from the laboratory environment to real world applications is often a difficult process. Envisioned applications proposed by scientists often meet significant translational barriers due to the immaturity of nanotechnology and the existence of a wide variety of competing nanomaterials with outstanding properties. There are also stringent requirements that need to be met in specific fields of applications before the end user will utilize new materials intended to replace the previous technologies. For example, penetration into the biomedical optical reagent market requires that the reagent be: (1) convenient to use by an end user from (at least no more difficult than the currently used reagent), (2) be validated both *in vitro* and *in vivo*, (3) and offer measurable and significant (rather than incremental) benefit to end users in specific applications. Moreover, significant and unambiguous documentation (protocols for use and quality control documentation) are often highly desired and standard in the biomedical reagent market. Adaption of FND for the bioimaging market involved significant prolonged efforts reflected in a number of reviews.^12,19,32,33,98,99^ Based on extensive *in vitro* and *in vivo* validations of FND applicability in bioimaging, it is expected that the material will start to be accepted by the biomedical community in the near future. In Sec. III A, we demonstrate a few validations performed by our group.

Another potentially impactful field of application of FDP containing negatively charged NV centers is nanometrology, which focuses on implementation of high precision measurements of environmental parameters such as electromagnetic field, temperature, and mechanical strain. We refer readers to recent excellent reviews in this field.^11,12,22,100^

The most recent and potentially highly disruptive application of FDP containing NV centers is related to hyperpolarization of ^13^C nuclei and external liquids for enhanced magnetic resonance imaging (MRI) contrast,^101–104^ or as direct MRI contrast agents.^105^ We review this application in more detail in Sec. III B. Finally, in Sec. III C, we discuss the FDP potential for nonmedical arena, specifically as tags for anticounterfeiting applications.

### A. *In vitro* and *in vivo* imaging

**Fig. 21.**
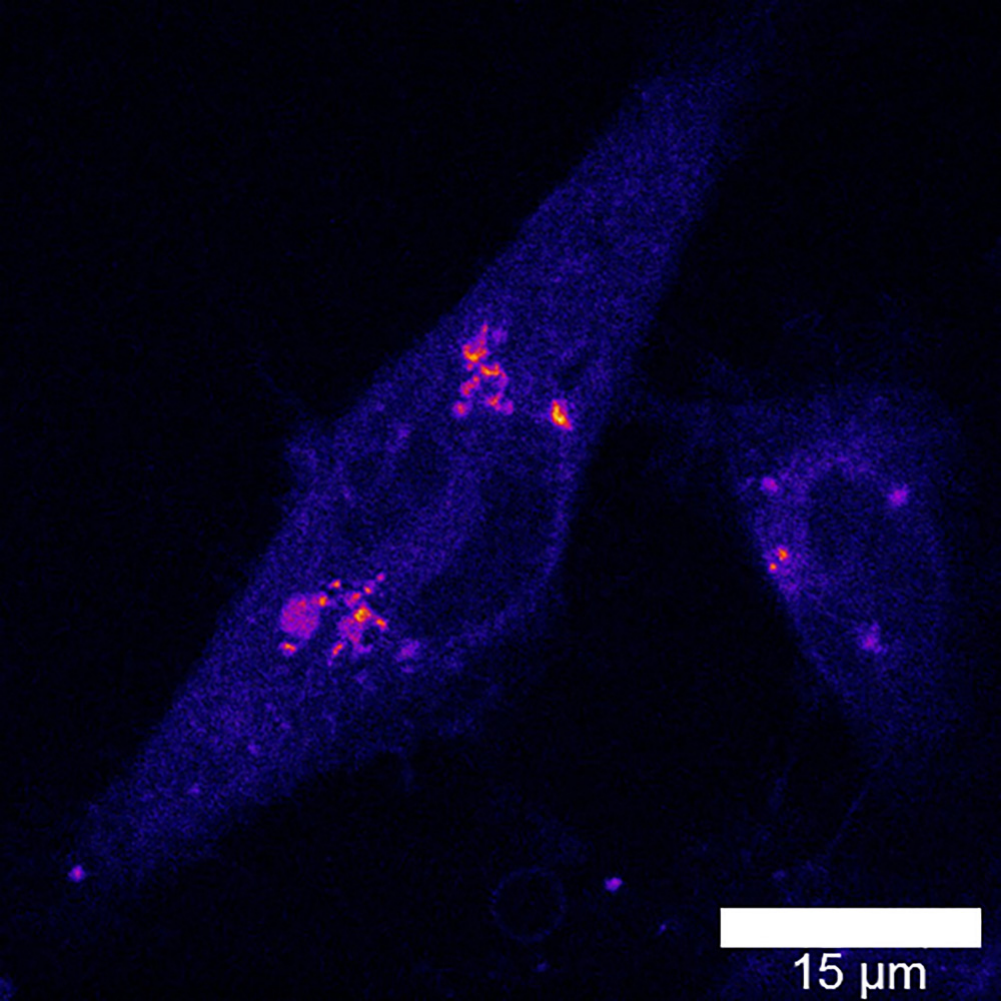
*In vitro* imaging of 20 nm FND in MDA-MB-231 Breast Cancer Cells with 488 nm laser excitation following 48 h incubation at 50 *μ*g/ml (650–720 nm detection window) (Image courtesy of N. Prabhakar, Åbo Akademi, Finland).

The frontiers of FND based imaging in biological environments has continued to advance over the years. As microscopy techniques mature, so do the quality of FND particles at smaller sizes, opening up new capabilities.^32^ For example, 30 nm particles allowed staining of E-selectin inflamed cells, increasing cellular detection by 40×.^106^ Even imaging of NV containing particles with sizes down to 20 nm has been readily demonstrated by laser excitation (Fig. 21), which several years ago was difficult to achieve.

**Fig. 22.**
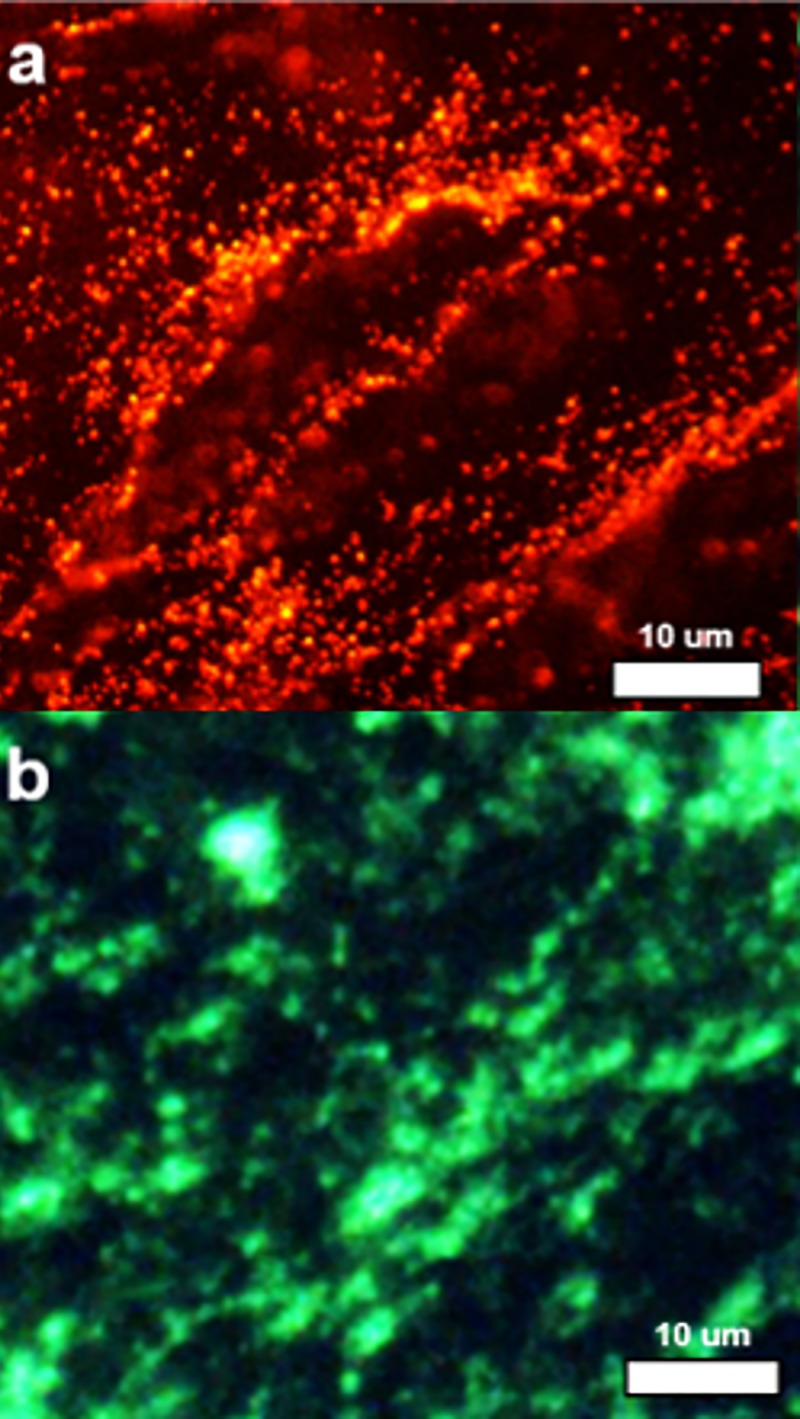
Labeling of activated platelets (GPIIb/IIIa) through *in vivo* clot model via (a) FND-Streptavidin + antiCD41-Biotin or (b) FITC-antiCD41 (Images courtesy of Dr. Arepally, Department of Hematology, Duke University).

In addition to small sizes being achieved, the utility of FND as a medical stain has been demonstrated, extending beyond function as just a simple research tool for labeling cellular cultures. Recently, diamond has been used to label blood clots, which invites the exciting potential of their use for a therapeutic application. There have been *in vitro* validations that particles of 1 *μ*m down to 200 nm are useful in selectively labeling clot formation via conjugation with bitistatin,^107^ both utilizing red (NV) and green (NVN) fluorescence.^108^ Figure 22 highlights that streptavidin functionalized FND at 100 nm can be used to label clots incubated with a CD41 antibody, selective for activated platelets, in a manner similar to FITC-antiCD41. Importantly, the long Stokes’ shift of FND provides contrast against background autofluorescence.

**Fig. 23.**
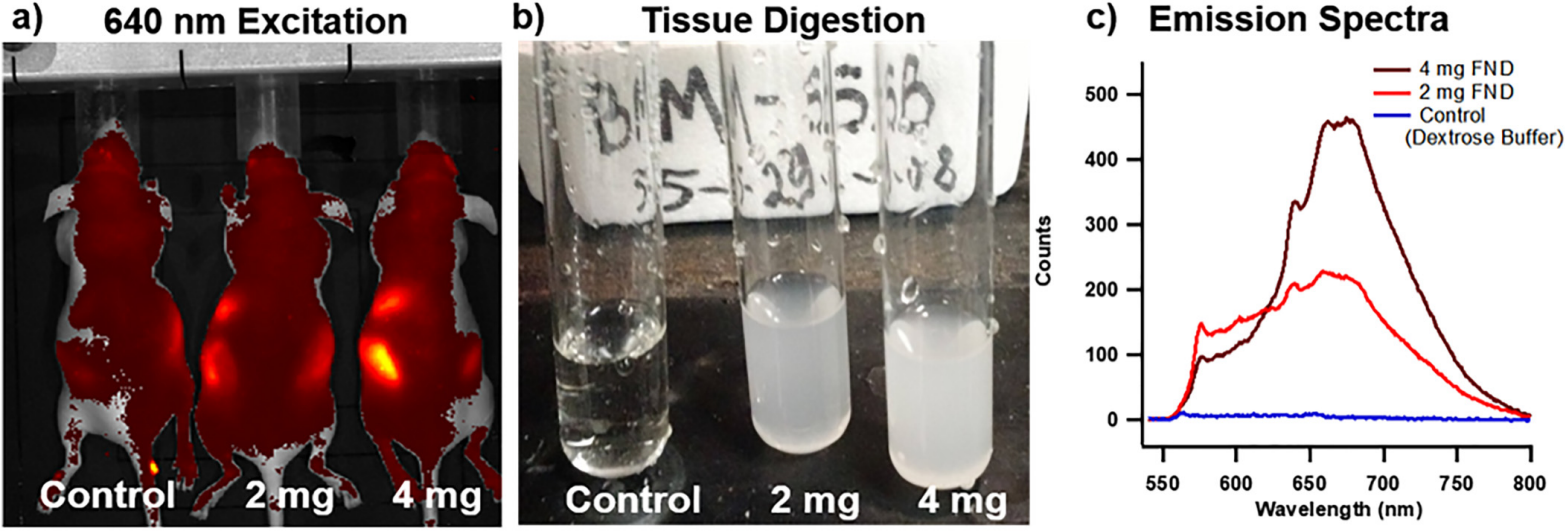
Whole-body IVIS imaging. (a) Images of three mice, administered 0, 2, or 4 mg/ml nanodiamond in 5% dextrose buffer (courtesy of Ashlyn Rickard, Palmer group, Department of Radiation Oncology, Duke University). (b) Spleens after piranha acid digestion, showing contrast of accumulated particles. (c) Bulk spectra of samples in 3 indicating dose-dependent recovery and validation of FND presence.

**Fig. 24.**
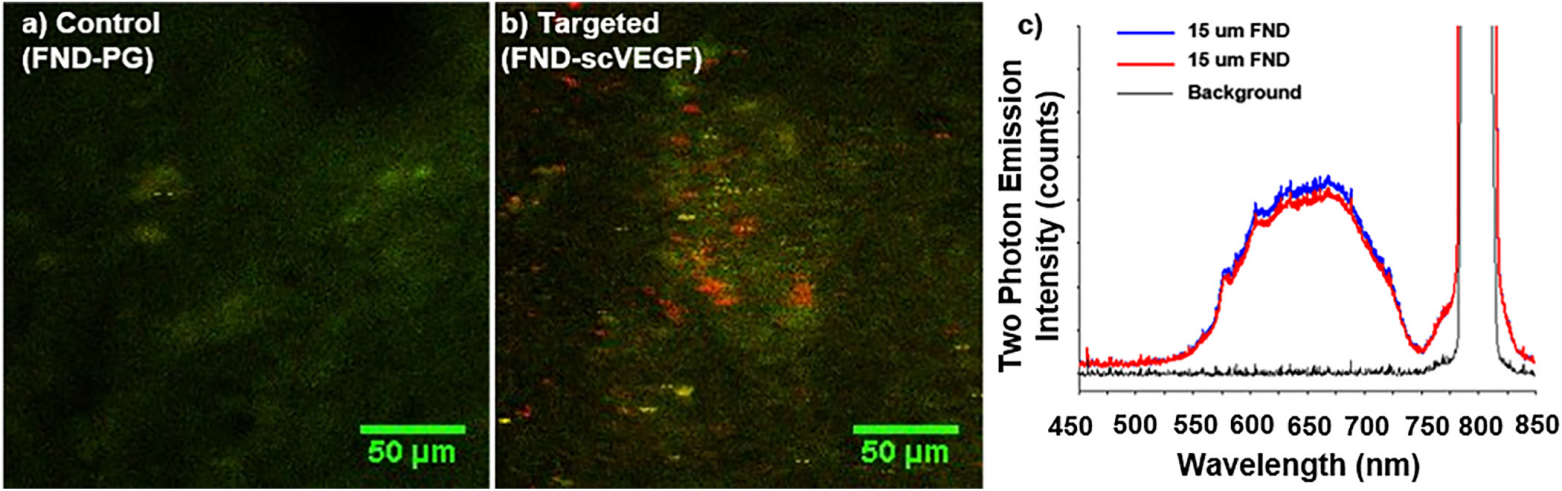
Two-photon imaging (810 nm excitation) of 10 *μ*m mouse tumor sections for (a) buffer administered and (b) fluorescent nanodiamond conjugated with scVEGF and administered to mice bearing 4T1 carcinomas; composite of green (500–550 nm) and orange/red (575–610 nm) emission. Microscope: Zeiss 7 MP with Chameleon Vision S excitation. (c) shows emission spectra for these particles with 800 nm excitation.

These complexities of FND extend into full animal imaging. The potential for *in vivo* imaging has been demonstrated, either using specialized methods of background subtraction to enhance constrast^109^ or by multiple example modes of injection.^110^ Recently, we demonstrated in a pilot study that ∼170 nm FND seems to be the threshold whereby whole-body imaging may be accomplished via an In Vivo Imaging System (IVIS, Perkin Elmer) system at sufficient concentration (Fig. 23). The IVIS system in an *in vivo* imaging system which allows for 2D optical imaging and 3D tomography of whole organisms. Here, FND could be recovered by tissue digestion in Pirhana acid solution (3:1 ratio of H_2_SO_4_:H_2_O_2_) and analyzed by fluorescence. Importantly for such *in vivo* work, there are an increasing number of studies that recognize the safety of FND for use *in vivo.*^111,112^ Microscopy techniques such as multiphoton imaging should have relevance to animal studies, allowing for increased spatial resolution at longer penetration depths. Figure 24 highlights the use of multiphoton imaging to detect FND in cryosections after targeting of tumors by vascular endothelial growth factor (a signaling protein that stimulates the formation of blood vessels) conjugation, with corresponding emission spectra for the particles with 800 nm excitation. Because photobleaching of traditional fluorophores is an issue in multiphoton imaging due to increased laser strength,^113^ FND as an alternative provides a clear advantage.

### B. Applications of hyperpolarized diamond particle

MRI plays an important role in research and clinical diagnostics and avoids the exposure of patients to ionizing radiation required by other methods.^114^ The MR technique is based on the measurement of interactions between spin active nuclei and radiofrequency waves after polarization in a strong magnetic field. Detection sensitivity of MR is based on the population difference between oppositely aligned nuclear spins in a magnetic field termed “polarization,” which is small for the majority of isotopes at thermal equilibrium.^115^ Standard MRI modalities operate based on relaxation properties of ^1^H nuclear spins, which are naturally abundant as water constituents in humans and biological organisms with near-unity isotopic abundance and which possess the highest gyromagnetic ratio (γ) of ∼4.3 kHz/G providing the highest signal per spin.^116^ ^1^H MRI is mostly used to gather information on the anatomical structure of soft tissues. In some instances, to enhance contrast in functional imaging (to measure physiological parameters in addition to anatomical structure) exogenous contrast agents [e.g., based on gadolinium or superparamagnetic iron oxide (SPIO)] are used to alter the relaxation time of nearby water protons, thereby altering the contrast in the image.^117^ Though carbon is the third most abundant element by atomic percent in biological organisms and could be an essential tracer in many important biological processes, it is very difficult to resolve its presence in the body using conventional MRI due to the low natural abundance of ^13^C spin (only 1.1%) and low γ (∼1.1 kHz/G) resulting in long acquisition times.^115^ For comparison, polarization at a magnetic field (B) strength of 3-Tesla for H and C nuclei is ∼0.01% and ∼0.00026%, correspondingly. Increasing the sensitivity of clinical MRI systems, and particularly ^13^C MRI, to reach higher sensitivity and enhanced contrast in molecular and metabolic imaging studies has thus been a long-sought goal.^115,118^

The advancement of hyperpolarization, which temporarily boosts MR signal intensities by 4–5 orders-of-magnitude beyond thermal equilibrium by redistributing the population of nuclear spins, has provided new opportunities for ^13^C MRI.^115,118^ This process typically uses very low temperature, high magnetic fields, and electromagnetic radiation to manipulate the nuclear spin population. For instance, the dynamic nuclear polarization (DNP) method first spin-polarizes an electron bath, which is easier to polarize at low temperature and then transfers this polarization to nearby nuclear spins through dipole interactions. Cancer diagnostics with hyperpolarized magnetic resonance imaging (HP MRI) provides a capability not attainable with previous MRI techniques. Using hyperpolarized ^13^C-enriched biomolecules active in specific cellular metabolism, HP MRI provides unique information on metabolic alterations of the imaging agents in real time. A dose-ranging study of HP [1-13C]pyruvate in patients with prostate cancer established the safety and feasibility of this technique, while additional studies are ongoing in prostate, brain, breast, liver, cervical, and ovarian cancer.^118^ The technology for generating hyperpolarized agents has advanced, and new MR data acquisition sequences and improved MRI hardware have been developed.^119^ In parallel, development of DNP-hyperpolarized nanoparticles based on ^13^C and ^29^Si as MRI contrast imaging agents has been also pursued.^116,120,121^ In general, nanoparticles have longer blood circulation times than molecular species and provide higher molar sensitivity. Nanoparticles have been used for decades as contrast agents in MRI and computed tomography (CT) imaging in preclinical animal cancer studies for tumor demarcation, perfusion, cell tracking in immune cell therapies and many other implementations.^122^ However, current HP MRI techniques have limitations. The available imaging time is restricted to roughly a minute at room temperature, due to the short nuclear polarization lifetime (T1) of ^13^C in organic compounds which limits the duration of metabolic imaging. Moreover, current hyperpolarization methods are expensive and restrictive, involving cryogenic temperatures, large magnetic fields, and extensive time requirements to hyperpolarize the molecular ^13^C based agents^118^ or particles of Si or diamond.^116,120^

Recently, it was shown that ^13^C polarization can be strongly enhanced within bulk diamond at room temperature in under a minute through optical pumping of NV color centers.^122^ In addition, hyperpolarization induced by optical pumping in bulk diamond is long-lasting (hours). Following exciting developments for bulk diamond, nanodiamonds emerged as a new paradigm for optical hyperpolarization that promises to reshape and significantly enhance classical NMR spectroscopy and imaging. This relies on the fact that the NV centers in these diamond particles [in the negative charge state (NV^−^)] can be optically polarized close to 100% even at room temperature and independent of magnetic field.^123^ This vastly athermal spin polarization can then be potentially transferred to nuclear spins (of an external liquid or biomolecules for instance) in contact with the high surface area particles, hyperpolarizing them through the Overhauser effect, and enhancing their NMR signature by orders of magnitude. The first method of optical ^13^C hyperpolarization in diamond particles was recently developed,^101^ showing their efficient polarization at low fields (1–70 mT), and high throughput (∼20 mg/min), and demonstrated the ability to construct low cost diamond hyperpolarizer devices that could retrofit existing NMR and MRI magnet systems.^124^ The ^13^C nuclei of large (200 *μ*m) diamond microparticles were optically hyperpolarized to the ∼0.75% level, a gain over their Boltzmann polarization at high field (7 T) by a factor of about 750, and a corresponding imaging acceleration time in MRI by over a million-fold.^123^

It is envisioned that optically hyperpolarized nanodiamonds (HP ND) can play two major roles in cancer HP MRI imaging: (i) as targeted ^13^C contrast agents to directly map malignancies and (ii) as a platform to hyperpolarize external molecules. Importantly, optical hyperpolarization, as opposed to the DNP approach, can be *replenished* under ambient conditions, highlighting the possibility of *in vivo* hyperpolarization of ND particles for continuous MRI (Ref. 101) and possibly polarization of surrounding molecules.^125^ The first niche application—injection of HP ND contrast agents directly for MRI imaging—has value for preclinical animal/cell/tissue studies such as tumor demarcation, cell tracking, and perfusion with sensitivity exceeding current MRI capabilities. Using HP ND as an external platform to hyperpolarize safe, ^13^C endogenous molecules at room temperature would significantly mitigate the cost of the current hyperpolarization techniques. These molecules (like pyruvate) would be subsequently injected for MRI, providing valuable physiologic (perfusion, flow, angiographic) information with better sensitivity/resolution and safer (than gadolinium) contrast agents, while also providing better metabolic information than radio-isotope positron emission tomography (PET) imaging. Additionally, hyperpolarization in diamond particles also opens the possibility of “dual-mode” imaging.^121^ Utilizing the nonbleaching bright fluorescence of NV centers for optical imaging in concert with MRI, where an intrinsically fluorescent MRI agent would dramatically facilitate histopathological analysis and assessment of biodistribution in post mortem analysis.

It is useful to provide a comparison to alternative existing and emerging deep tissue imaging techniques in terms of sensitivity and resolution. Along with MRI, the x-ray based CT is used for anatomical imaging and PET is used for functional imaging. MRI has lower sensitivity as compared to PET, while the resolution is higher.^126^ MRI and CT have similar resolution. As opposed to these two technologies, MRI does not expose personnel and patients to ionizing radiation. A recently emerged magnetic particle imaging (MPI) method based on magnetic particles like SPIO (Ref. 117) is a promising technology currently used in preclinical studies. MPI’s resolution is comparable to what is expected for HP ND, but magnetic particles have much higher sensitivity. The combination of MPI and other anatomical imaging modalities (e.g., CT) can allow for comprehensive imaging of anatomical features.^127^ Imaging using HP ND on the contrary will be compatible with ^13^C MRI instrumentation used for metabolic imaging and ^1^H MRI used for anatomical imaging without the additional burden of equipment cost.^118^

These exciting recent results highlight diamond as a unique material with the potential to shift the paradigm for an established medical diagnostic tool.

### C. Potential industrial applications

Industrial applications of FDP have only begun to emerge. One potential interesting area of application is as anticounterfeiting tags. As supply chains become more complex due to globalization, with parts coming from a diverse set of suppliers, identifying counterfeit products has become a major challenge.^128^ Numerous approaches have been employed to provide a unique signature including imprinting designs, adding colorants, luminescent markers, or complex optical image systems, utilizing radio frequency identification tags, and a vast array of alternatives.^129^ Many organic colorants or luminescent markers tend to fade with time or to exposure to UV radiation as well as being susceptible to high temperature. Quantum dots often contain heavy metals which are to be avoided. The color centers in FDP are protected by the surrounding monocrystalline diamond, which make them inert/stable under a wide range of harsh conditions. So, the color centers are not affected by temperatures, pressures, chemical environments, or processes used in labeling and packaging or even in electronic device fabrication or mounting. FDPs have unique emission signatures which are virtually impossible to reverse engineer without knowing the processing history of the sample (irradiation with high-energy particles, annealing, etc.). Below we outline several observations which can guide the design of the FDP-based tags.

**Fig. 25.**
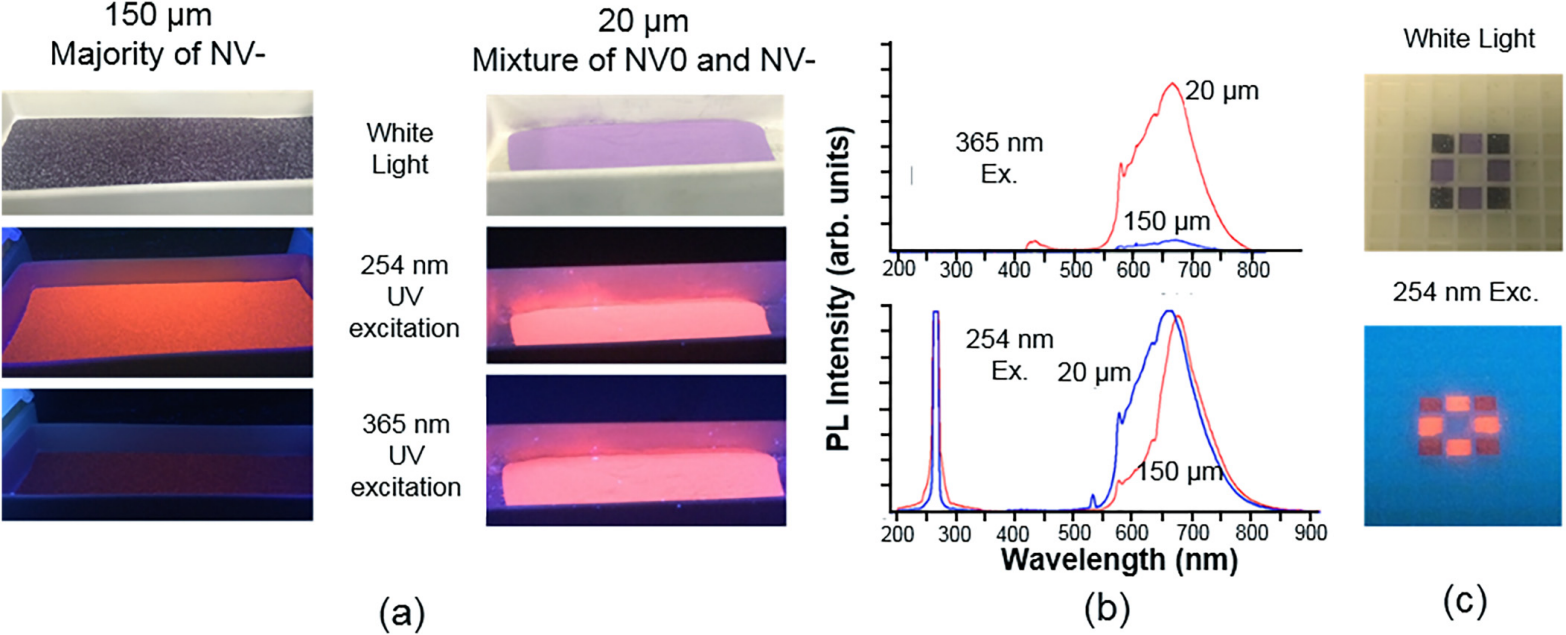
Photographs of diamond particles with a prevalence of NV^−^ centers (150 *μ*m) and a mixture of NV^0^ and NV^−^ centers (20 *μ*m) shown in white light and under 365 and 254 nm excitation produced by a UV lamp (a). The spectra of these samples are shown taken under 365 and 254 nm excitation light. (b) A pattern made from these two types of particles under white light and 254 nm UV excitation (c).

FDPs with unique spectral characteristics under UV excitation can be produced based on control of the ratio between neutral and negatively charged NV color centers. Stable, nonbleaching excitation of red emission under UV exposure is an important property for authentication tags. Even more important is emission that depends on the UV wavelength. We observed strikingly different behavior under UV excitation for type Ib diamond powders with approximately the same total N content from two different vendors following similar irradiation and annealing treatments (Fig. 25). Both demonstrated bright red emission under 254 nm excitation (SWUV), while under 365 nm excitation (LWUV), one of the powders was very weakly fluorescent. Spectra are presented in Fig. 25, providing a quantitative picture of the emitted light. The powder active only under 254 nm UV was high quality as-grown 150 *μ*m diamond particles, while the powder active at both SWUV and LWUV was 20 *μ*m powder obtained by crushing of as-grown diamond grits from a different vendor. Note the difference in emitted color (and peak position in the spectra) of the powders under 254 nm excitation, red/orange versus pink/orange for the 150 and 20 *μ*m particles, respectively.

From a thorough analysis of the samples, we concluded that the observed phenomena originate from the fact that the as-grown sample contains a predominance of NV^−^ centers and only a small amount of NV^0^. In contrast, the 20 *μ*m crushed sample contains an appreciable amount of both NV^0^ and NV^−^ centers. Based on the excitation spectra reported in the literature for these defect centers,^1^ NV^0^ can be excited by both SWUV and LWUV, but NV^−^ cannot be excited by either (or only very weakly by LWUV). Instead, excitation of NV^−^ under SWUV and LWUV can only be achieved via reabsorption of the emission from NV^0^ centers. The small amount of NV^0^ in the 150 *μ*m sample is not enough to provide bright emission when excited by 365 nm UV, nor is it enough to provide significant amounts of light for reabsorption-excitation of NV^−^ centers; hence, the sample appears relatively dark. At 254 nm, very strong absorption from NV^0^ centers is expected since this wavelength is close to the bandgap energy for this defect center. Hence, very strong emission occurs, and both 150 and 20 *μ*m appear very bright. Since the emission from the small amount of NV^0^ present in the 150 *μ*m sample is so strong, it is sufficient to provide light for reabsorption-excitation of NV^−^ centers. In addition to this striking behavior, it was also observed that an *inverted ZPL* peak at 637 nm (NV^−^) is present under UV excitation due to self-absorption (Fig. 25) of neighboring NV^−^ centers at 637 nm as the absorption/emission lines at this wavelength overlap. This inversion of the ZPL peak was always observed when taking spectra of powders, and never when taking spectra of dispersed particles. These novel identifying characteristics are nearly impossible to reverse engineer without a deep knowledge of the starting material, processing and associated mechanisms of the excitation/emission.

**Fig. 26.**
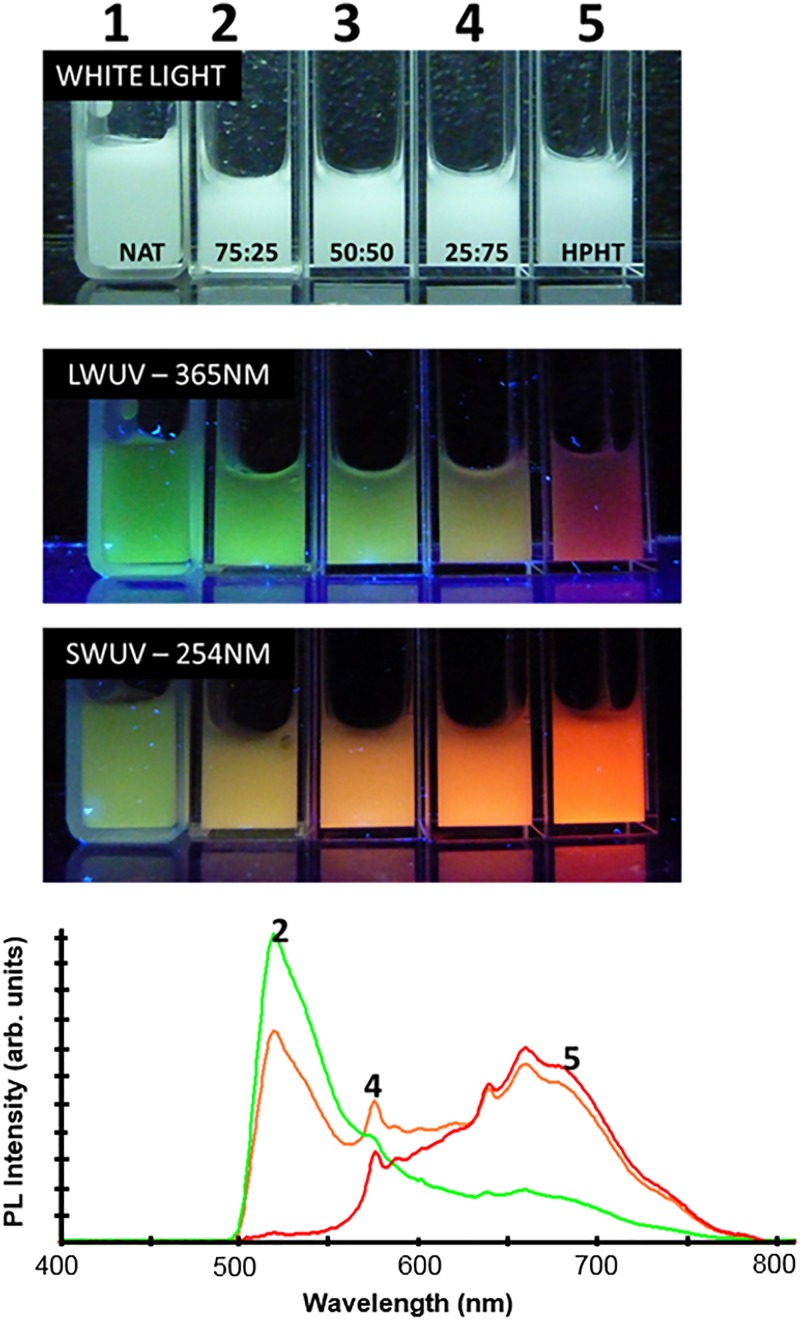
Mixtures of 1 *μ*m FDP containing NV centers (#5) and H3 centers (#1) and PL spectra of three samples.

Figure 26 illustrates particles with H3 centers and NV centers. Mixtures of particles in certain proportions result in very distinctive color signatures under LWUV and SWUV irradiation. Spectral signatures are also shown for a few samples under blue excitation. This approach indicates that the “true” signature (e.g., NV) can be obscured in background noise, but can be extracted visually using specific excitation, filters or spectroscopically.

Based even on these few examples, it is obvious that a wide range of implementations of FDP in tagging and identification is possible. Chosen particles can be small to not be detected by unaided eye or large enough so that only one individual particle (∼40 *μ*m in size) can be used for a single label (emission is strong enough for detection). FDP tags may be employed surreptitiously on a surface, buried in a matrix or both, as well as incorporated into various materials during processing or added as a tag at the end of fabrication.

## IV. CONCLUSIONS

Diamond is a material with a very rare combination of properties, starting with unmatched physical properties such as strength, hardness, thermal conductivity, and extending to outstanding optical properties including luminescent centers emitting in the UV, visible, and IR spectral regions with exceptional photostability even at high temperature. However, when it comes to engineering luminescent centers of interest, many of diamond’s exceptional properties become significant barriers toward production of luminescent diamond particles, especially particles at the nanoscale. Therefore, commercialization of this technology posts high entry barriers involving development of complex technological steps such as use of high-energy irradiation, milling/fragmentation of diamond, purification with strong etchers/oxidizers from milled media, and surface functionalization with diverse types of surface groups for meeting requirements for specific applications. Many centers require production of vacancies which need ∼40 eV energy to knock a carbon atom out of its lattice site. Vacancies are produced by irradiation with high-energy particles, e.g., 1–10 MeV electron beams, available only at dedicated facilities. Production of hundred-gram batches of the material is necessary to make it economically viable. Use of such powerful irradiation introduces a significant damage to the particles and often irreversible graphitization if a heat sink is not properly implemented. The yield of particles surviving irradiation strongly depends on the particle size, with much higher yield for particles tens of microns in size. However, during sequential milling of these particles to nanosized fractions, the yield of sub-50 nm particles is below ∼10%–20%. Thus, optimization of the complex parameter space is required for production, which involves production of FDP of different sizes for various applications. Finding applications for all the sizes produced is required to minimize the end user cost.

One important observation was made following experiments conducted to optimize the fluorescence brightness of diamond particles containing NV centers based upon irradiation fluence-dependent generation of NV centers (Sec. II B). Intuitively, it was assumed that a higher irradiation fluence producing a higher density of NV centers would result in the highest brightness.^31,50^ However, in fluence-dependent electron irradiation experiments,^60^ it was observed that for lower irradiation fluences (below approximately 5 × 10^18^ e^−^/cm^2^) the density of NV centers and associated photoluminescent brightness increases linearly with fluence, while at higher fluences, the NV density grows at a much slower rate and comes to saturation at a fluence of approximately 5 × 10^19^ e^−^/cm^2^ (the associated cost for this fluence is tens of thousands of USD in a typical irradiation facility). Surprisingly, fluorescence brightness begins to degrade with fluence after reaching a maximum brightness despite further increase of the density of NV centers. This degradation is partially associated with the introduction of parasitic defects with increasing fluence (such as W16, W33, etc.^60^) and partially with fluorescence quenching at high densities of defects, as was observed in natural diamonds.^1,2^ Thus, “over-irradiated” particles are both costly and have lower brightness. These observations allowed us to decrease the production cost of FDP by irradiating particles to the optimum fluence and creating the optimal density of NV centers. The optimal fluence/density of NV centers depends on particle size. During milling, though, the density of NV centers significantly decreases due to their degradation by introducing lattice distortions. As a result, for particles with sizes below ∼50 nm produced from optimized micron-sized particles, the maximum density of NV centers is ∼1–2 ppm (Sec. II B 2 c).

Another point that requires further development is a source of diamond for production of FDP. Currently, synthetic HPHT Ib diamond particulate with ∼100 ppm of substitutional N, which seems to be close to the optimal N content. Higher N content leads to increased density of NV but with much lower fluorescence possibly to the light absorption by N complexes (see Sec. II B 2 e). It would be desirable to conduct experiments with HPHT diamond with even lower than 100 ppm N content to further optimize the N content. Currently available synthetic HPHT diamond powder was optimized for the abrasives industry and have one serious drawback—nonuniform N distribution within the volume of the particles resulting in nanosized particles with high nonuniformity of brightness (see Fig. 15). One approach to overcome this barrier is bottom-up growth of FDP by either HPHT or CVD methods with controlled N (or other dopants) content in the carbon precursor or in the stock gas (Sec. II A). These methods, however, are in their infancy and the FDP production scale-up will be the next challenge after feasibility has been demonstrated. Stepping into the field of large manufacturers of commercial HPHT diamond growth and controlling the N uniformity in the particles might be a possible solution of this problem if the market size of FDP reaches a value that will raise the interest of large HPHT diamond producers.

Based on current users of fluorescent diamond particles, an array of luminescent centers is of interest including NV (both with negative and neutral charge states), H3, N3, SiV, and NE8. Thus, producing FDP with a varied color palette is important for FDP to be competitive with other fluorophores which are available in a wide range of luminescent colors. Novel method of rapid annealing of irradiated HPHT diamond particles at temperatures exceeding ∼1700 °C (Ref. 97) opens opportunities for the commercial production of diamonds with blue, green, and red emission, as well as controlled production of diamond with a mixture of the colors (Sec. II C).

One major focus of FND applications has centered around bioimaging and biosensing. In the field of bioimaging, there is still the unmet need of exceptionally bright, photostable, nontoxic particles *of a few nanometers in size* with emission in red/NIR.^19^ At the moment, it is doubtful that FND can satisfy this “ideal biolabel” requirement, especially in light of the highly overcrowded domain and appearance of other candidates such as organic fluorophores with improved stability, quantum dots, upconverting particles, etc.^19^ Thus, key opportunities for development revolve around improving the quality of NV centers in particulate diamond, especially at sizes <30 nm. While a number of groups,^12,32,83–85^ including ours,^32^ have improved the quality of these quantum fluorescent diamond particles, it is generally accepted that the quality needed has not yet been obtained. Larger size FND with high brightness still are considered in applications such as fiducial markers (above ∼40 nm FND) or imaging agents for mapping cancerous liaisons in preclinical studies where long term observations are required.^20,21^ Industrial, nonmedical applications of FDP in anticounterfeiting, forensic, and solar cells might be areas which can generate high demand of the material. Some of the potentially most impactful applications of fluorescent diamonds are predicated on the unique combination of optical and spin properties of the NV^−^ center. For instance, the use of NV containing particles for enhanced NMR spectroscopy,^130,131^ hyperpolarization of 13C nuclei and external liquids for enhanced MRI contrast,^101–104^ or as direct MRI contrast agents themselves^105^ are under development as potentially highly disruptive technologies.
